# CITE-Seq Analysis Reveals a Differential Natural Killer Cell *SPON2* Expression in Cardiovascular Disease Patients Impacted by Human-Cytomegalovirus Serostatus and Diabetes

**DOI:** 10.3390/ijms26031369

**Published:** 2025-02-06

**Authors:** Sujit Silas Armstrong, Daniel G. Chen, Sunil Kumar, James R. Heath, Matthew J. Feinstein, John R. Greenland, Daniel R. Calabrese, Lewis L. Lanier, Klaus Ley, Avishai Shemesh

**Affiliations:** 1 La Jolla Institute for Immunology, La Jolla, CA 92037, USA; 2 Institute of Systems Biology, University of Washington, Seattle, WA 98109, USA; daniel.chen@isbscience.org (D.G.C.);; 3 Immunology Center of Georgia, Medical College of Georgia, Augusta, GA 30912, USA; 4 Parker Institute for Cancer Immunotherapy, San Francisco, CA 94129, USA; lewis.lanier@ucsf.edu; 5 Division of Cardiology, Department of Medicine, Northwestern University Feinberg School of Medicine, Chicago, IL 60611, USA; 6 Department of Medicine, University of California, San Francisco, CA 94143, USA; 7 Medical Service, VA Health Care System, San Francisco, CA 94121, USA; 8 Department of Microbiology and Immunology, University of California, San Francisco, CA 94143, USA

**Keywords:** natural killer cells, *SPON2*, cardiovascular disease, cytomegalovirus, diabetes

## Abstract

Coronary artery disease (CAD) is linked to atherosclerosis plaque formation. In pro-inflammatory conditions, human Natural Killer (NK) cell frequencies in blood or plaque decrease; however, NK cells are underexplored in CAD pathogenesis, inflammatory mechanisms, and CAD comorbidities, such as human cytomegalovirus (HCMV) infection and diabetes. Analysis of PBMC CITE-seq data from sixty-one CAD patients revealed higher blood NK cell *SPON2* expression in CAD patients with higher stenosis severity. Conversely, NK cell *SPON2* expression was lower in pro-inflammatory atherosclerosis plaque tissue with an enriched adaptive NK cell gene signature. In CAD patients with higher stenosis severity, peripheral blood NK cell *SPON2* expression was lower in patients with high HCMV-induced adaptive NK cell frequencies and corresponded to lower PBMC *TGFβ* transcript expression with dependency on diabetes status. These results suggest that high NK cell *SPON2* expression is linked to atherosclerosis pro-homeostatic status and may have diagnostic and prognostic implications in cardiovascular disease.

## 1. Introduction

Coronary artery disease (CAD) represents a third of all cardiovascular diseases (CVDs) and is associated with atherosclerotic plaque formation in the arteries that supply blood to the heart, leading to acute coronary syndrome (ACS) and myocardial infarction (MI) [1,2,3]. Elevated interferon gamma (IFNγ) levels are known to be linked with pro-inflammatory responses and atherosclerosis plaque vulnerability due to arterial stenosis suppression [2]. This vulnerability can result in plaque rupture and thrombosis, responsible for most ACS cases [3,4]. Plaque growth and constrictive remodeling contribute to stenosis formation, which may lead to plaque stabilization or angina [5,6,7]. Stenosis severity alone does not reflect the inflammatory status or predict ACS risk, as chronic stenosis and plaque growth also correlate with plaque healing [4,8]. Higher levels of transforming growth factor beta (TGFβ), a potent pro-hemostatic, pro-fibrotic, and anti-inflammatory cytokine, is associated with stability of atherosclerotic plaques [9,10,11,12,13]. Among CAD comorbidities, diabetes increases the atherosclerosis burden and constrictive remodeling [14,15], although the mechanisms are not fully understood. Other key risk factors for CAD include human cytomegalovirus (HCMV) infection [16], obesity, increased low-density lipoprotein (LDL) cholesterol blood levels, and high blood pressure [17]. HCMV infection is positively correlated with the level of the inflammatory marker high-sensitivity C-reactive protein (hs-CRP) and low-grade inflammation, which increases the risk of ACS [3,18].

NK cells are innate lymphocytes that regulate immune, non-immune, virally infected, and transformed cells by cytokine secretion, such as IFNγ, and direct cell lysis, which are suppressed by TGFβ [19,20,21]. NK cells are CD3^−^CD56^+^CD16^−/+^ lymphocytes with two main subsets in peripheral blood: immature CD56^bright^CD16^−^CD57^−^ and mature CD56^dim^CD16^+^CD57^+/−^ cells. Mature NK cells can further be subdivided into subsets by differential expression of NKG2A and CD57 [22]. HCMV-induced adaptive NK cells, characterized as CD56^dim^CD16^+^NKG2A^−^NKG2C^high^, can be further subdivided by the presence or absence of the adaptor protein FcεR1γ, coded by the *FCER1G* gene [22]. Adaptive CD56^dim^CD16^+^NKG2A^−^NKG2C^high^FcεR1γ^−^ NK cells (g-NK cells) exhibit reduced expression of the IL-2 receptor beta-chain (*CD122*, *IL2RB*), PLZF (*ZBTB16*), and EAT-2 (*SH2D1B*) and higher expression of *IL-32*, *CCL5*, *GZMH*, *BCL11B*, *KLRC3* (NKG2E), and *LAG3* [22,23,24,25]. This signature is also reported in KLRC2-deficient humans, indicating other mechanisms of adaptive differentiation besides NKG2C expression, such as by CD16 and CD2 stimulation [26]. Lower NKG2C expression is also found in NKG2A^+^CD57^−^ immature and NKG2A^+/−^ mature NK cells from HCMV seronegative individuals [22,23,24,25,26,27]. Furthermore, interleukin-15 (IL-15) can promote, and TGFβ can suppress, FcεR1γ upregulation in human NK cells, leading to an adaptive-like NK cell phenotype [22].

In CAD, lower human peripheral blood NK cell frequencies correlate with low-grade cardiac inflammation, and NK cells are more activated in ACS [28,29,30]. Additionally, lower NK cell frequencies are observed in pro-inflammatory carotid plaques relative to stable pro-homeostatic femoral plaques [31]. In a clinical study of coronary atherosclerosis, higher peripheral blood adaptive NKG2C^+^CD57^+^ NK cell frequencies were associated with lower plaque volume in young individuals and an overall lower relative risk of coronary atherosclerosis, suggesting a protective role [32]. By contrast, others have reported that NKG2C^+^ NK cells are associated with plaque instability [33]. Overall, there are limited data regarding the human NK cell gene expression profiles and changes in frequencies of NK cell subsets in CAD and atherosclerosis and the influence of other comorbidities such as HCMV, diabetes, or inflammation status [28,29,30,34].

Here, we investigated peripheral blood NK cell gene expression and NK cell subsets in PBMC CITE-seq data from a CAD cohort of sixty-one patients [35] stratified by stenosis severity and diabetes and HCMV serostatus. To validate our observations, we further analyzed scRNA-seq data of plaque NK cells from patients with atherosclerotic plaques [31]. As adaptive NKG2C^+^CD57^+^ NK cells are associated with lower plaque volume and plaque instability, we hypothesized that a mature non-adaptive NK cell gene signature or subset frequencies would increase with the patients’ pro-homeostatic status. This could suggest a mechanism of plaque growth and constrictive remodeling or plaque healing [36], with potential diagnostic and therapeutic implications. We found that in CAD patients with severe stenosis, peripheral blood NK cells expressed higher levels of *SPON2*, a gene coding for Spondin-2 (Mindin), a secreted extracellular matrix protein [37,38], and known to be increase in the plasma of cardiovascular disease patients [39]. The increase in *SPON2* expression was associated with lower HCMV-induced NK cell frequencies and a relatively higher PBMC *TGFβ* transcript expression, suggesting a connection to a pro-homeostatic and/or pro-fibrotic state in cardiovascular disease.

## 2. Results

### 2.1. NK Cell SPON2 Expression Increases with CAD Stenosis Severity

**Figure 1:**
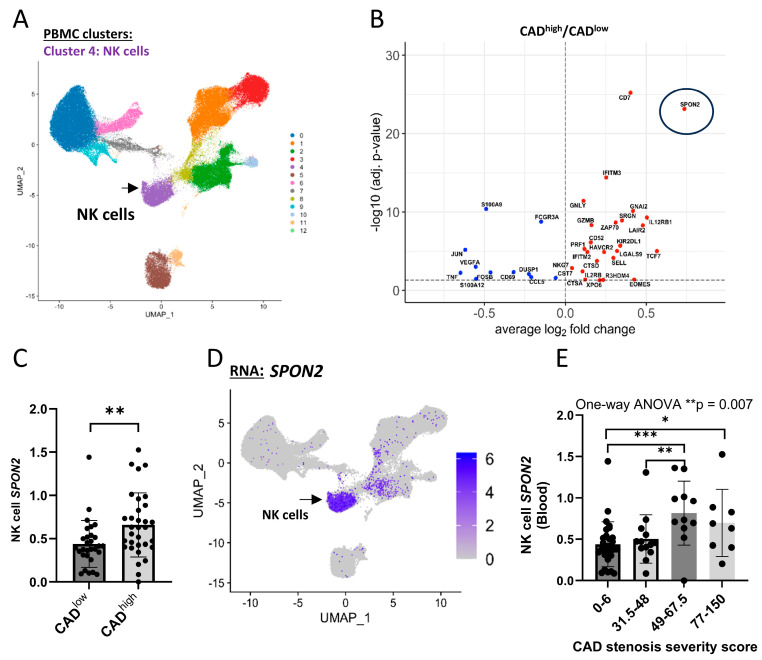
NK cell *SPON2* expression significantly increases in CAD patients with high stenosis. (**A**) PBMC from 61 CAD patients were clustered by CITE-seq protein expression. UMAP of PBMC clusters based on CITE-seq protein expression. NK cells (purple cluster, black arrow). (**B**) Differential gene expression (DGE) analysis of NK cell gene expression between CAD^low^ vs. CAD^high^ at the single-cell level (red: upregulated in CAD^high^; blue: upregulated in CAD^low^). (**C**) Patients’ mean NK cell *SPON2* expression in CAD^low^ vs. CAD^high^ patients (dot = patient). (**D**) UMAP of *SPON2* RNA expression relative to PBMC clusters. (**E**) NK cell *SPON2* expression in patients grouped by CAD stenosis severity score. Patients were grouped the patients by stenosis severity score (combined percent stenosis of each artery segment score): I, 0–6 [*n* = 29, CAD^low^]; II, 30–48 [*n* = 13]; III, 49–67.5 [*n* = 11]; IV, 77–150 [*n* = 8]. Mean +/− S.D.; Mann–Whitney test; one-tail; * *p* < 0.05; ** *p* < 0.01; *** *p* < 0.001.

To study the relationship between CAD and NK cells in humans, we analyzed PBMC CITE-seq data from a cohort of sixty-one patients with no prior events of ACS or MI (asymptomatic) and diagnosed with a low or high stenosis (percent stenosis of each artery segment: CAD^low^ as 0–6 and CAD^high^ as >30; GSE190570; Supplementary Table S1) [35]. To identify blood NK cells, we used CITE-seq surface protein expression [40] and identified 12 immune cell clusters (Figure 1A). Cluster 4 (>10,000 cells) was characterized as surface CD56^+^, CD16^+/−^, CD3^−^, CD19^−^, CD20^−^, CD14^−^, CD123^−^, and CD4^−^ cells (Figure S1A). Furthermore, cluster 4 cells expressed high levels of NK cell-related genes: *NKG7* (Natural Killer cell granule protein 7), *PRF1* (perforin), *GZMB* (granzyme B), *KLRG1* (KLRG1), *IL2RB* (CD122), and *TBX21* (T-bet) (Figure S1B). As these peripheral blood CD3^−^CD56^+^ and CD16^+/−^ cells expressed protein *PRF1* and *KLRG1* mRNA, we designated this cluster as NK cells [41]. To identify a CAD-specific phenotype for NK cells, we performed a differential gene expression analysis between CAD^low^ vs. CAD^high^ patients at the single-cell level (Figure 1B). One of the genes expressed at higher levels in NK cells from CAD^high^ patients was *SPON2*. The patients’ mean NK cell *SPON2* expression significantly increased in CAD^high^ patients (Figure 1C). NK cell *SPON2* expression did not change relative to other clinical parameters (Figure S1C). We also observed that *SPON2* was strongly expressed by the NK cell cluster relative to the CD4 or CD8 T cell clusters (Figure 1D and Figure S1A). To examine if *SPON2* expression increased with stenosis severity, we grouped the patients by stenosis severity score (combined percent stenosis of each artery segment score; Supplementary Table S2). Patients with a higher stenosis severity displayed a significant increase in *SPON2* expression in their peripheral blood NK cells (Figure 1E and Figure S1D). These results indicate an increase in NK cell *SPON2* expression with stenosis severity in CAD patients.

### 2.2. NK Cell SPON2 Expression Is Lower in Pro-Inflammatory Atherosclerosis Plaque Tissue

**Figure 2:**
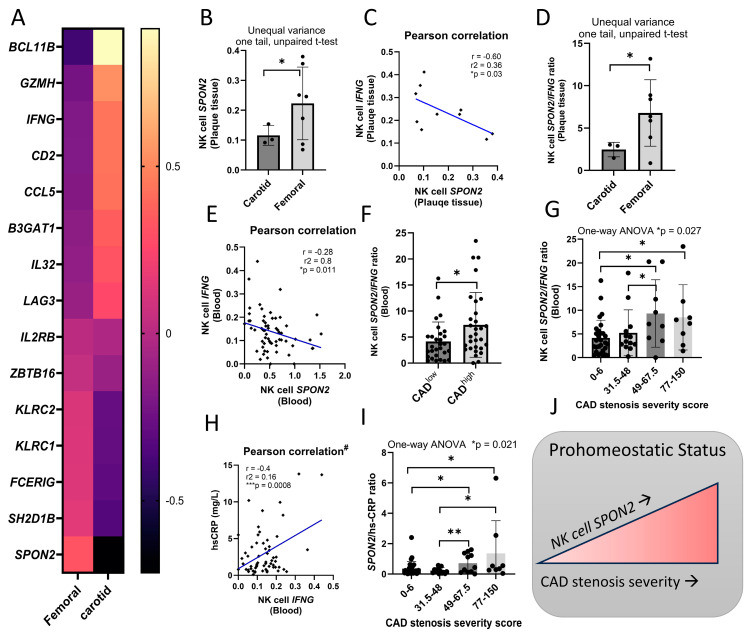
NK cell *SPON2* expression is higher in pro-homeostatic plaque tissue. (**A**) NK cell gene expression between carotid plaques (pro-inflammatory, *n* =3) relative to the femoral plaques (pro-homeostatic, *n* =7)**.** Gene expression is displayed as mean z-score. (**B**) Mean NK cell *SPON2* expression in femoral relative to carotid plaque tissue. (**C**) Pearson correlation (one-tail) between NK cell *SPON2* and *IFNG* expression in atherosclerosis plaques (*n* = 10). (**D**) NK cell *SPON2*/*IFNG* ratio in carotid or femoral plaques. (**E**) Pearson correlation (one-tail) between NK cell *SPON2* and *IFNG* expression in CAD patients. NK cell *SPON2*/*IFNG* ratio in (**F**) CAD^low^ vs. CAD^high^ patients or (**G**) relative to stenosis severity. (**H**) Pearson correlation (one-tail) between NK cell *IFNG* expression and hs-CRP levels in CAD patients. ^#^ To avoid misinterpretation of the data, one patient outlier (hs-CRP (mg/L) = 150) was removed from the analysis. (**I**) NK cell *SPON2*/ hs-CRP ratio relative to stenosis severity. (**J**) Schematic representation of findings. Mean +/− S.D.; Mann–Whitney test; one-tail; * *p* < 0.05; ** *p* < 0.01; dot = patient.

Higher IFNγ and adaptive NKG2C^+^ NK cells are associated with plaque instability [2,30,33]. To investigate the clinical relevance of *SPON2* in NK cells more specifically with relation to atherosclerosis, we examined *SPON2* expression in pro-inflammatory carotid vs. pro-homeostatic femoral plaque tissue [31], in relation to *IFNG* and adaptive NK cell gene signature (Figure 2A). We found higher *SPON2* expression in plaque NK cells in femoral pro-homeostatic plaques (Figure 2A,B). In contrast, in the pro-inflammatory carotid plaques, we found higher NK cell *IFNG* expression and enriched adaptive NK cell gene signature (e.g., higher *BCL11B*, *GZMH*, *IL32*, *CCL5*, *B3GAT1*, and *LAG3* and lower *KLRC1*, *FCERIG*, and *SH2D1B*). Plaque NK cell *SPON2* expression negatively correlated with NK cell *IFNG* expression (Figure 2C). The ratio between *SPON2* and *IFNG* increased in femoral plaques (unpaired t-test; one-tail; unequal variance [* *p* = 0.013]; Figure 2D). This suggested that higher NK cell *IFNG* expression and lower *SPON2* expression are associated with pro-inflammatory status.

We then investigated *SPON2* expression relative to *IFNG* expression in blood NK cells in CAD patients. Blood NK cell *SPON2* expression negatively correlated with *IFNG* (Figure 2E), and a higher *SPON2*/*IFNG* ratio was associated with lower *SPON2* expression in CAD^high^ (severe stenosis) patients (Figure 2F,G). Moreover, *IFNG* displayed a positive correlation to hs-CRP (mg/L) (Figure 2H), and the *SPON2*/hs-CRP ratio significantly increased relative to stenosis severity (Figure 2I), while blood NK cell frequencies did not differ relative to CAD status or stenosis (Figure S1E). These results suggest that higher peripheral blood NK cell *SPON2* expression in asymptomatic CAD patients is associated with reduced NK cell *IFNG* expression and a pro-homeostatic relative to pro-inflammatory status.

### 2.3. SPON2 Expression Decrease in HCMV-Seropositive Diabetes-Negative Patients

**Figure 3:**
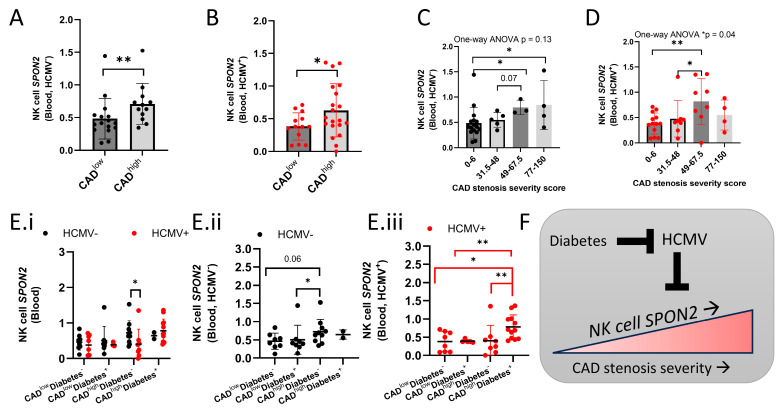
HCMV serostatus and diabetes impact on NK cell *SPON2* expression. Mean NK cell *SPON2* expression per patient grouped by CAD^low^ vs. CAD^high^; (**A**) HCMV seronegative (HCMV^−^) or (**B**) HCMV seropositive (HCMV^+^). NK cell *SPON2* expression relative to stenosis severity in (**C**) HCMV^−^ or (**D**) HCMV^+^ patients. (**E**) NK cell *SPON2* expression in patients, grouped by CAD, diabetes, and HCMV status (black: HCMV^−^; red: HCMV^+^); (**E.i**) all patients, (**E.ii**) HCMV^−^ patients, or (**E.iii**) HCMV^+^ patients. (**F**) Schematic representation of figure findings. Mean+/− S.D.; Mann–Whitney test; one-tail; * *p* < 0.05; ** *p* < 0.01; dot = patient.

HCMV is associated with increased pro-inflammation and higher risk of ACS [3,18]. Out of the sixty-one CAD patients, thirty-three patients were HCMV seropositive (HCMV^+^). As we observed lower *SPON2* expression in plaque tissue with an enriched adaptive NK cell gene signature, we addressed if *SPON2* expression in CAD is negatively impacted by HCMV status. Therefore, we examined *SPON2* expression in relation to HCMV status (HCMV^−^ or HCMV^+^) and CAD status (CAD^low^ vs. CAD^high^). *SPON2* expression significantly increased in the NK cells of HCMV^−^ or HCMV^+^ CAD^high^ patients, yet HCMV^+^ patients displayed a less significant increase (Figure 3A,B) suggesting HCMV-induced *SPON2* suppression. Relative to stenosis severity, NK cells from HCMV^+^ patients displayed a more significant increase in *SPON2* expression, suggesting an additional mechanism of regulation (Figure 3C,D).

Diabetes is associated with plaque constrictive remodeling [14,15]. Out of the sixty-one CAD patients, twenty-seven patients were diagnosed with T2DM (Type 2 diabetes [Diabetes]; Supplementary Table S3). Therefore, we addressed if diabetes has a positive impact on NK cell *SPON2* expression relative to CAD and HCMV status at the patient level [I: CAD^low^diabetes^−^ (*n* = 16), II: CAD^low^diabetes^+^ (*n* = 13), III: CAD^high^diabetes^−^ (*n* = 18), and IV: CAD^high^diabetes^+^ (*n* = 14)]. The CAD^high^diabetes^+^ group displayed a higher relative number of HCMV^+^ cases (85.71% relative to 50%, 38.46%, and 44.44%, respectively; Supplementary Table S3), which was age-independent and was not associated with high systolic blood pressure (BP) levels, BMI score, or other clinical parameters (Figure S1F). *SPON2* expression displayed no differences in diabetic HCMV^+^ CAD^high^ patients relative to HCMV^−^ patients (Figure 3E.i). In contrast, *SPON2* expression decreased in NK cells from non-diabetic HCMV^+^ CAD^high^ relative to HCMV^−^ patients (Figure 3(E.i)) and displayed a significant increase in non-diabetic HCMV^−^ CAD^high^ relative to CAD^low^ patients, which was not detected in the HCMV^+^ patients (Figure 3(E.ii,E.iii)). The data indicate an HCMV-seropositive-induced restriction of *SPON2* expression and diabetes suppression of HCMV-seropositive-induced influence (Figure 3F).

### 2.4. SPON2^low^ HCMV-Induced FcεR1γ^−/low^ NK Cell Frequencies Persist with Stenosis Severity

**Figure 4:**
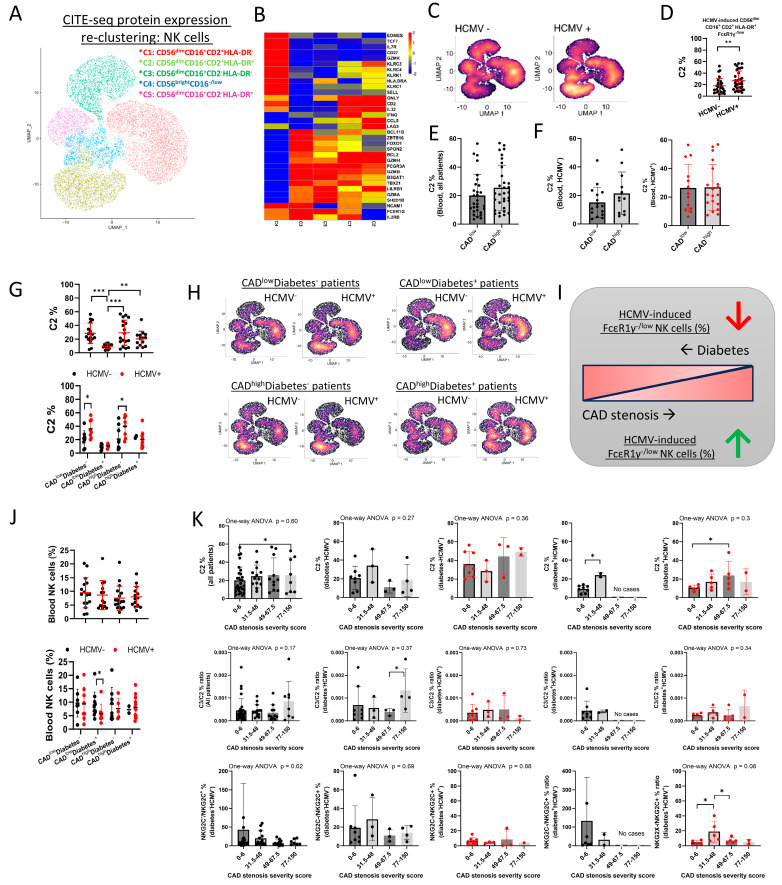
*SPON2^low^* HCMV-induced NK cell frequencies do not decrease with stenosis severity. (**A**) UMAP of NK cell clusters. The NK cell cluster (Cluster 4, Figure 1A) was re-clustered based on CITE-seq protein expression of CD56, CD16, CD25, CD27, CD2, or HLA-DR. (**B**) Heatmap of mean NK cell gene expression for the indicated genes. (**C**) density plots of NK cell clusters in HCMV^−^ or HCMV^+^ patients. C2 frequencies (%) between (**D**) HCMV^−^ and HCMV^+^ patients, or patients grouped by (**E**) CAD low vs. high status, (**F**) CAD and HCMV serostatus, or (**G**) CAD, diabetes, and HCMV status. (**H**) Density plot of NK cell clusters in patients grouped by CAD, diabetes, and HCMV status. (**I**) Schematic representation of findings. (**J**) Blood NK cell frequencies in patients grouped by CAD, diabetes, and HCMV status. (**K**) C2 frequencies (%) (upper panels), C3/C2 ratio (middle panels), or NKG2C^−^/NKG2C^+^ ratio (lower panels) relative to stenosis severity. Patient groups (not all patients) are shown as black: HCMV^−^ or red: HCMV^+^). Mean+/− S.D.; Mann–Whitney test; one-tail; * *p* < 0.05; ** *p* < 0.01; *** *p* < 0.001; dot = patient.

HCMV infection can lead to adaptive NK cell differentiation [22,23,24,25,26,27]. *SPON2* expression has previously been reported in non-adaptive NK cells [42], and our results indicate HCMV^+^ status limits *SPON2* expression. Therefore, we addressed if the higher NK cell *SPON2* expression in CAD^high^ relative to CAD^low^ patients is due to lower frequencies of HCMV-induced adaptive NK cells. Hence, we sub-clustered the NK cell cluster to identify NK cell subsets. As CD2 is known to play a critical role in *KLRC2*-deficient adaptive NK cells [26,43], and adaptive NK cells are reported to express higher HLA-DR [44,45] (Figure S2A), we sub-clustered the NK cells based on CITE-seq protein expression of CD56, CD16, CD2, HLA-DR, and the immature associated markers CD27 and CD25 [22] (Figure 4A and Figure S2A,B). We identified five NK cell sub-clusters (C1–5). C4 displayed an immature CD56^bright^CD16^−/low^, CD25^+^, CD27^+^, CD2^+^, and HLA-DR^+^ phenotype. C1, C2, C3, and C5 displayed a mature CD56^dim^CD16^+^ phenotype, with CD2^+^HLA-DR^+^ (C2) or HLA-DR^−^ (C1), and CD2^−/low^HLA-DR^+^ (C5) or HLA-DR^−^ (C3) (Figure S2B).

In line with other publications [24,25,26,42,46], gene expression analysis of NK cell maturation and adaptive-associated genes characterized C1 and C2 with high *IFNG*, *FCGR3A* (CD16), *CD2*, *IL32*, *LAG3*, *CCL5*, *GZMH*, *BCL2*, *KLRC3*, *KLRC4*, and *GNLY*, with lower expression of *SPON2*, *ZBTB16* (PLZF), *SH2D1B* (EAT-2), *FCERIG* (FcεR1γ), *GZMK*, and *CD27*, and intermediate expression of *GZMA* and *GZMB*, indicating these clusters have an enriched mature or adaptive-NK cell gene signature. Relative to C1, C2 expressed lower *SPON2*, *IL2RB*, *ZBTB16*, *SH2D1B*, *FOXO1*, *TBX21*, and *FCERIG* and higher *LAG3*, *HLA-DRA* (HLA-DR), *CCL5*, *KLRC3*, and *KLRC4*, suggesting this cluster is enriched with mature adaptive FcεR1γ^−/low^ NK cells or g-NK cells (Figure 4B). Furthermore, *HLA-DRA*, *CD2*, *IL32*, *LAG3*, and *KLRC3* were detected in the C4 cluster. Yet, C4 displayed a lower expression of *SPON2*, *FCGR3A*, *B3GAT1 (*CD57*)*, *GZMA*, *GZMH*, *GZMB*, *BCL11B*, and *LILRB1* (CD85J) and a higher expression of *NCAM1*, *KLRC1* (NKG2A), *EOMES*, *SELL*, *TCF7*, *IL7R*, *CD27*, and *GZMK* relative to the other four clusters, indicating the C4 is enriched with an immature NK cell gene signature (Figure 4B and Figure S2A,B). C3 and C5 displayed higher *SPON2*, *FCGR3A* (CD16), *GZMA*, *GZMB*, *ZBTB16* (PLZF), SH*2D1B* (EAT-2), and *FCERIG* (FcεR1γ), intermediate *BCL2* and GZMH expression, and lower *KLRC3*, *KLRC4*, *CD2*, *IL32*, *LAG3*, *SELL* (CD62L*)*, *TCF7* (TCF1), *IL7R*, *GZMK*, and *CD27* expression, suggesting an enriched mature non-adaptive NK cell gene signature (Figure 4B). C5 differed from C3 by lower expression of *SPON2*, *NCAM1* (CD56), *BCL2*, *TBX21*, and *BCL11B* and higher *HLA-DRA* (Figure 4B). Note that *KLRC2* (NKG2C) and anti-NKG2C antibody were not in the scRNA-seq BD Rapsody or CITE-seq panels, and the decrease in *KLRC1* (NKG2A) expression in the mature NK cell clusters corresponds to an enriched NKG2A^−^CD57^+^ NK cell subset in all patients, as confirmed with an independent Cytek analysis of matched PBMC samples (Figure 4B and Figure S3A).

*SPON2* expression was significantly lower in the C2 cluster relative to other mature NK cell clusters and showed a negative correlation to C2 frequencies and a positive correlation to C3 frequencies (Figure 4B and Figure S2C). Frequency comparison of the NK cell clusters relative to HCMV serostatus revealed a significant increase in C2 frequencies in HCMV^+^ patients (Figure 4C,D), while all other clusters did not show significant changes (Figure S4A), further indicating the C2 cluster is enriched for HCMV-induced adaptive NK cells. C2 frequencies did not decrease with CAD^high^ status (Figure 4E,F) but did decrease in diabetic CAD^low^ patients (Figure 4G–I), while an opposite trend was detected for C1 and C3 frequencies (Figure S4B–F). In line, diabetic patients displayed lower adaptive NKG2C^+^ frequencies. (Figure S3A). Similarly to C2 frequencies, blood NK cell frequencies were lower in diabetic HCMV^+^ CAD^low^ patients but not in diabetic HCMV^+^ CAD^high^ patients (Figure 4J). C2 frequencies, C3/C2 ratio, or NKG2C^−^/NKG2C^+^ ratio did not reveal significant changes relative to stenosis severity and diabetes or HCMV status (Figure 4K and Figure S5A). *SPON2* expression increased relative to stenosis severity in all NK cell clusters (Figure S5B). These results suggest the following: (1) diabetes suppresses C2 frequencies; (2) CAD stenosis suppresses diabetes’ impact on C2 frequencies; and (3) lower C2 frequencies do not explain the increase in *SPON2* expression relative to stenosis severity (systemic relative to cluster-specific influence).

### 2.5. Higher HCMV-Induced FcεR1γ^−/low^ NK Cell Frequencies Associate with Lower NK Cell SPON2 and PBMC TGFβ Transcript Expression

**Figure 5:**
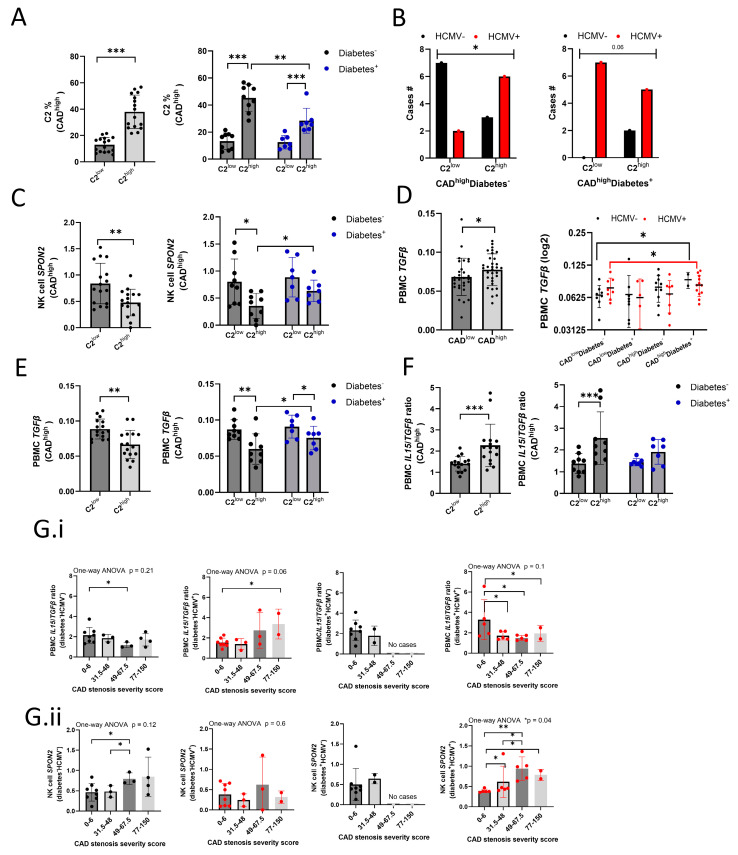
Higher NK cell *SPON2* expression corresponds to lower C2 frequencies and higher PBMC *TGFβ* expression in CAD^high^ patients. (**A**) C2 frequencies in CAD^high^ patients with low (C2^low^) or high (C2^high^) frequencies (left), and relative to diabetes status (right: black, diabetes^−^; blue, diabetes^+^). (**B**) Number of HCMV^−^ or HCMV^+^ cases in C2^low^ vs. C2^high^ patients: left, diabetes^−^; right, diabetes^+^. (**C**) Mean NK cell *SPON2* expression in CAD^high^ C2^low^ or C2^high^ patients (left), and relative to diabetes status (right: black, diabetes^−^; blue, diabetes^+^). (**D**) PBMC *TGFβ* expression in CAD low vs. high patients. Right: expression of PBMC *TGFβ* in patients grouped by CAD, diabetes, and HCMV status, or (**E**) in CAD^high^ C2^low^ or C2^high^ patients, and relative to diabetes status (right: black, diabetes^−^; blue, diabetes^+^). (**F**) PBMC *IL15*/*TGFβ* ratio in CAD^high^ C2^low^ or C2^high^ patients (left), and relative to diabetes status (right: black, diabetes^−^; blue, diabetes^+^). (**G**) PBMC *IL15*/*TGFβ* ratio (**G.i**) or mean NK cell *SPON2* expression (**G.ii**), relative to stenosis severity in (left to right) diabetes^−^HCMV^−^, diabetes^−^HCMV^+^, diabetes^+^HCMV^−^, or diabetes^+^HCMV^+^ patients. Patient groups are shown as black: HCMV^−^ or red: HCMV^+^). Mean +/− S.D.; Mann–Whitney test; one-tail; * *p* < 0.05; ** *p* < 0.01; *** *p* < 0.001; dot = patient.

Higher adaptive NK cell frequencies are known to be caused by with HCMV infection or reactivation [22,47] and are associated with plaque instability [30,33], whereas TGFβ is known to be associated with plaque stabilization and lower pro-inflammation [9,10,11,12,13]. We addressed if CAD^high^ patients, grouped by low or high C2 frequencies (C2^low^: 5.47–21.53%, *n* = 16; C2^high^: 21.63–56.75%, *n* = 16), and relative to diabetes status (C2^low^ or C2^high^: diabetes^−^, *n* = 9; diabetes^+^, *n* = 7) (Figure 5A,B), display lower NK cell *SPON2* expression and lower PBMC *TGFβ* transcript levels.

In line with our previous observations, non-diabetic C2^high^ patients displayed a significantly lower *SPON2* expression, which was higher in diabetic C2^high^ patients (Figure 5C). The decrease in *SPON2* expression was detected across all NK cell clusters (Figure S6A), indicating the lower *SPON2* expression is not only due to high C2 frequencies. C2^high^ patients did not show lower stenosis severity (Figure S6B) but displayed lower blood NK cell frequencies (Figure S6C), higher NK cell *IFNG* expression (Figure S6D), and a lower *SPON2*/*IFNG* ratio (Figure S6E), suggesting an increased pro-inflammatory status in the C2^high^ patients. In line with CAD status, we detected higher PBMC *TGFβ* transcript expression in CAD^high^ relative to CAD^low^ patients (Figure 5D). Non-diabetic CAD^high^C2^high^ patients displayed lower PBMC *TGFβ* expression and an increased expression in diabetic patients (Figure 5E). This observation suggests a relative increase in pro-inflammatory status in the non-diabetic CAD^high^C2^high^ patient group, attenuated by diabetes. These results show that higher C2 frequencies in CAD^high^ patients are associated with (1) lower *SPON2* expression, (2) lower blood NK cell frequencies, and (3) lower PBMC *TGFβ* transcript levels. Furthermore, the results demonstrate that, in diabetic patients, C2 frequencies are lower, and, accordingly, the decrease in *SPON2* expression and PBMC *TGFβ* transcript expression are attenuated. Therefore, this suggests an opposing relationship between HCMV-induced adaptive NK cells and NK cell *SPON2* expression and PBMC *TGFβ* expression.

TGFβ inhibits the positive influence of interleukin-2 (IL-2), IL-15, or interleukin-12 (IL-12) on NK cell activation and IFNγ secretion [21]. Therefore, we examined PBMC *IL2*, *IL15*, or *IL12* transcript expression. We did not detect *IL12* transcripts or significant variations in *IL2* transcript expression. Diabetic patients displayed a significant increase in *IL15* transcript expression (Figure S6F), which was associated with higher C2 frequencies in CAD^high^ patients. Examination of *TGFβ* expression relative to *IL15* expression (*IL15/TGFβ* ratio) revealed a higher *IL15/TGFβ* ratio in diabetic CAD^low^, but not in diabetic CAD^high^, patients (Figure S6G), which could explain the variations between C2 and C3 frequencies we observed in these groups (Figure S6H). The non-diabetic C2^high^CAD^high^ patients displayed a higher *IL15*/*TGFβ* ratio than C2^low^ patients, which was attenuated in diabetic patients (Figure 5F). The *IL15*/*TGFβ* ratio decreased with stenosis severity in diabetes^−^HCMV^−^, diabetes^+^HCMV^−^, and diabetes^+^HCMV^+^, but not in diabetes^−^HCMV^+^, patients (Figure 5(G.i)) 4, and NK cell *SPON2* expression displayed an opposite trend (Figure 5(G.ii)). This shows higher NK cell *SPON2* expression occurs in a TGFβ “rich” NK cell suppressive microenvironment and diabetes is associated with higher PBMC *IL15* and *TGFβ* expression in CAD patients.

### 2.6. IL-15 and TGFβ Differently Regulate Spondin-2 Expression in Adaptive and Non-Adaptive Primary NK Cells

**Figure 6:**
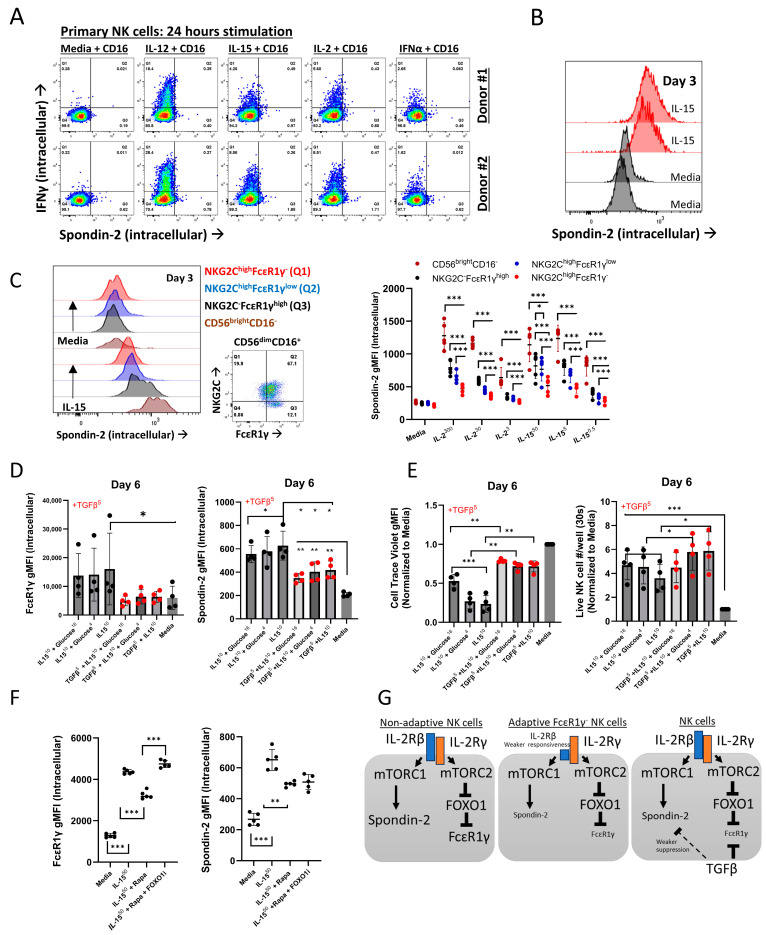
Differential upregulation of Spondin-2 by IL-15 and TGFβ in primary NK cells. (**A**) Dot plots of intracellular Spondin-2 (X-axis) vs. IFNγ (Y-axis) expression in isolated human primary NK cells (*n* = 2) following 24 h of CD16 stimulation with media without cytokines or with IL-12 (1 ng/mL), IL-2 (300 U/mL), IL-15 (50 ng/mL), or IFNα (50 ng/mL). (**B**) Histograms of Spondin-2 intracellular expression (red) relative to media without IL-15 control (black) in isolated primary NK cells following 3 days of stimulation with IL-15 (50 ng/mL). (**C**) Histograms of Spondin-2 intracellular expression between the defined NK cell subsets (color-coded) after 3 days of stimulation with IL-15 (50 ng/mL) or media without cytokines. The right dot plot represents the gating of mature CD56^dim^CD16^+^NK cells to identify adaptive NK cell subsets by NKG2C vs. FcεR1γ protein expression. Right panel: IL-2 or IL-15 concentration-dependent expression of Spondin-2 between the defined NK cell subsets (*n* = 5). IL-2 (300, 30, and 3 U/mL), IL-15 (50 and 0.5 ng/mL), or media (media without cytokines). (**D**) FcεR1γ (left) or Spondin-2 (right) intracellular expression or (**E**) cell trace violet (left) or relative NK cell numbers/well (right) in purified primary non-adaptive NK cells (*n* = 4) stimulated for 6 days with IL-15 (10 ng/mL), with or without glucose (16 or 4 g/L) or TGFβ (5 ng/mL) relative to media without cytokines. (**F**) Expression of FcεR1γ (left) or Spondin-2 (right) in purified primary non-adaptive NK cells stimulated for 6 days with IL-15 (50 ng/mL) with or without rapamycin (RAPA, 10 nM) or FOXO1 inhibitor (50 nM) relative to media without cytokines. (**G**) Schematic representation of findings. Mean+/− S.D.; Mann–Whitney test; one-tail; * *p* < 0.05; ** *p* < 0.01; *** *p* < 0.001; dot = donor.

We investigated Spondin-2 upregulation in primary NK cells downstream of IL-2, IL-15, and IL-12 stimulation. Short (1 day) IL-2, IL-15, or IL-12 and CD16 cross-linking stimulation of primary NK cells that induced IFNγ expression did not lead to Spondin-2 expression (Figure 6A), indicating a differential regulation between IFNγ and Spondin-2 expression. Spondin-2 upregulation was detected in IL-15-stimulated primary NK cells on day 3 (Figure 6B), indicating that Spondin-2 upregulation occurs downstream of the IL-2/IL-15 receptor.

We then compared Spondin-2 upregulation between immature (CD56^bright^ CD16^−^), mature (CD56^dim^ CD16^+^), non-adaptive NKG2C^−^FcεR1γ^high^, and adaptive NKG2C^high^FcεR1γ^low^ or NKG2C^high^FcεR1γ^−^ NK cells on day 3 (Figure 6C). Either IL-2 or IL-15 led to Sponidn-2 upregulation in a concentration-dependent manner. Adaptive NKG2C^high^FcεR1γ^−^ or FcεR1γ^low^ expressed lower Spondin-2 protein relative to other NK cell subsets. The results indicate a differential Spondin-2 upregulation in non-adaptive vs. adaptive NK cell subsets downstream of the IL-2/-15 receptor, which can explain our observation of lower *SPON2* expression in the C2 cluster and C2^high^ patients. This suggests that a higher adaptive FcεR1γ^−/low^ NK cell frequency might limit *SPON2* increase due to their lower responsiveness to IL-2 or IL-15.

To further investigate Spondin-2 upregulation in the context of diabetes and TGFβ, we stimulated purified NK cells with IL-15 (10 ng/mL), with or without TGFβ (5 ng/mL) and in the presence of higher glucose concentrations (16 or 4 g/L relative to culture media) and measured Spondin-2 upregulation, or FcεR1γ upregulation as a positive control, at day 6. In line with our prior study [22], TGFβ completely suppressed FcεR1γ upregulation and cell proliferation induced by IL-15, while the increased glucose concentrations showed a negative influence on FcεR1γ upregulation and cell proliferation (Figure 6D,E). In contrast, TGFβ partly suppressed Spondin-2 upregulation and sustained NK cell numbers during IL-15 stimulation (Figure 6D,E and Figure S6E), and glucose had a negative influence on Spondin-2 upregulation and cell proliferation.

These results show a differential regulation of Spondin-2 expression relative to FcεR1γ by TGFβ. Therefore, we examined Spondin-2 upregulation during mTOR inhibition by rapamycin (RAPA) and co-inhibition of FOXO1 [22]. As expected, FcεR1γ upregulation was suppressed by rapamycin and was salvaged by FOXO1 co-inhibition (Figure 6F) [22]. In contrast, rapamycin suppressed Spondin-2 upregulation, whereas FOXO1 co-inhibition did not salvage Spondin-2 upregulation, thus validating a mechanism of differential regulation of Spondin-2 relative to FcεR1γ (Figure 6G). These results show that Spondin-2 can be expressed by NK cells in the presence of TGFβ, and TGFβ can sustain NK cell numbers during NK cell activation and can explain the increase in NK cell *SPON2* expression and sustained NK cell percentages and C2 frequencies in CAD^high^ patients.

## 3. Discussion

Here, we show that *SPON2* expression in human blood NK cells increases with stenosis severity in CAD patients. Moreover, NK cell *SPON2* levels decreased in pro-inflammatory relative to pro-homeostatic atherosclerosis plaques, with the former showing an enriched adaptive NK cell gene signature. In CAD patients with higher stenosis, higher *SPON2* expression corresponds to higher PBMC *TGFβ* transcript expression and lower HCMV-induced adaptive NK cell frequencies. These results suggest that NK cell *SPON2* expression is associated with a pro-homeostatic status. In humans, Spondin-2 plasma levels can increase with cardiovascular disease [39], and Spondin-2 is downregulated in humans with failing hearts [48]. Yet, the role of Spondin-2 in atherosclerosis in humans is not defined. In mice, *Spon2* knockout increases cardiac risk [48,49,50]. Atherosclerosis plaque tissue status can be defined as pro-inflammatory with higher plaque vulnerability and risk of rupture or pro-homeostatic with higher stability and reconstructive remodeling [31]. Our findings show that the increase in NK cell *SPON2* levels is associated with pro-homeostatic status. Thus, blood NK cell *SPON2* expression might have a value in diagnosis, prognosis, and therapeutical intervention in atherosclerosis. Still, additional research is needed to investigate if NK cell *SPON2* expression has a direct function on plaque vulnerability or stability or atherosclerosis outcomes such as acute coronary syndrome, myocardial infarction, or angina.

Adaptive NK cells are associated with increased pro-inflammation and higher plaque instability [30,33]. Our findings support the association of adaptive NK cells with pro-inflammatory conditions which might lead to plaque rupture. Thus, high frequencies of adaptive NK cells might be a risk factor in CAD and atherosclerosis, and these patients might be monitored for plaque rupture and acute coronary syndrome or other outcomes, such as strokes [32]. Still, a limitation of our study is that all CAD patients were asymptomatic. Thus, further longitudinal research studies are required to evaluate adaptive NK cells as a risk factor in symptomatic relative to asymptomatic patients.

Our findings also indicate that the increase in *SPON2* expression is suppressed in CAD^high^C2^high^ patients but not only due to higher frequencies of HCMV-induced adaptive NK cells with lower IL-15 responsiveness. Interestingly, in our study, C2^high^CAD^high^ patients had lower PBMC *TGFβ* transcript expression. We have previously shown that the interplay between IL-15 and TGFβ stimulation during CD16 cross-linking (NK cell activation) leads to an adaptive-like NK cell phenotype by suppressing NK cell proliferation [22]. This can explain some of our results regarding variations in the frequencies of NK cell clusters in the CAD^low^ or CAD^high^ diabetic patients due to changes in their PBMC *IL15*/*TGFβ* ratio. Still, C2^high^CAD^high^ patients express a relatively lower PBMC *TGFβ* transcript expression. This can suggest a direct suppression of adaptive NK cells on *TGFβ* transcript expression. HCMV promotes TGFβ secretion in infected cells [51]. This can suggest an alternative mechanism for higher plaque vulnerability associated with NKG2C^+^ NK cell activation and lysis of TGFβ expressing HCMV infected cells. Accordingly, as diabetes suppressed HCMV-induced adaptive NK cell frequencies, it enhanced PBMC *TGFβ* transcript and NK cell *SPON2* expression. This suggests a TGFβ-dependent mechanism of plaque remodeling by diabetes through suppression of adaptive NK cell frequencies and upregulation of *SPON2* expression.

Overall, our results reveal the relationship of CAD and its comorbidities, diabetes, and HCMV infection, with human NK cells. These observations suggest that higher NK cell *SPON2* expression is associated with pro-homeostatic status, higher *TGFβ* transcript expression, and lower adaptive NK cell frequencies, which might have a value in early diagnosis, prognosis, and therapeutic intervention in atherosclerosis and cardiovascular disease.

## 4. Materials and Methods

### 4.1. Sample Collection and Quantitative Coronary Angiography (QCA) Quantification

As was previously described, individuals between 40 and 80 years old suspected of having coronary artery disease were recruited from the Coronary Assessment in Virginia cohort (CAVA) through the Cardiac Catheterization Laboratory at the University of Virginia Health System, Charlottesville, VA, USA. Written informed consent was obtained from all participants, and the study received approval from the Human Institutional Review Board (IRB No. 15328). Peripheral blood samples were collected from these participants before catheterization. Patients underwent standard cardiac catheterization with specific views of the coronary arteries. QCA was performed using automatic edge detection to analyze various parameters related to stenosis, including minimum lumen diameter, reference diameter, percentage diameter stenosis, and stenosis length. Analysis was carried out by experienced investigators who were blinded to the study. The severity score was determined based on the percentage stenosis of each artery segment, and the scores were combined to determine the overall angiographic disease burden. Patients were classified as CAD high if their score was >30 and CAD low if their score was <6. Diabetes status was evaluated by hemoglobin A1c (HbA1C) percentage and blood glucose (mg/dL) levels. Patients were grouped by stenosis severity score (combined percent stenosis of each artery segment score): I, 0–6 [*n* = 29, CADlow]; II, 30–48 [*n* = 13]; III, 49–67.5 [*n* = 11]; IV, 77–150 [*n* = 8]. Blood samples were collected before the SARS-CoV-2 outbreak. None of the patients reported having prior heart failure, and all patients exhibited a normal creatinine range, indicating normal kidney function [52,53].

### 4.2. Preparation of PBMC Samples

Peripheral blood samples were collected from coronary artery disease patients and individuals who underwent cardiac catheterization to exclude CAD. PBMCs were isolated from the blood samples using Ficoll-Paque PLUS (GE Healthcare Biosciences AB, Uppsala, Sweden) gradient centrifugation. Cell viability was assessed using Trypan blue staining, and the PBMCs were cryopreserved in a freezing solution (90% fetal bovine serum with 10% DMSO). Prior to analysis, the frozen PBMCs were thawed, and the viability and cell count were determined. The tubes containing the PBMCs were centrifuged at 400× *g* for 5 min, and the cells were then resuspended in a combination of 51 AbSeq antibodies, with each antibody added at a volume of 2 μL and 20 μL of BD’s Stain Buffer solution. This resuspension process was performed on ice for 30–60 min according to the manufacturer’s recommendations. Afterward, the cells were washed and counted once again. Out of the total 65 samples examined, 61 samples successfully passed the quality control assessment with a cell viability rate exceeding 80%. For each subject, the cells were tagged using a Sample Multiplexing Kit from BD Biosciences, Franklin Lakes, NJ, USA. The kit included oligonucleotide cell labeling. The tagged cells were subsequently washed three times, mixed, counted, stained with the relevant antibody mix, washed three more times, and, finally, loaded into Rhapsody nano-well plates. Each plate accommodated four samples.

### 4.3. Library Preparation and Single-Cell RNA-Sequencing

Pre-sequencing quality control (QC) was conducted using Agilent TapeStation high-sensitivity D1000 screentape. Each tube was cleaned using AMPure XP beads and was washed with 80% ethanol. The cDNA was then eluted, and a second Tapestation QC was performed, followed by dilution as necessary. The samples were combined into a pool and subjected to sequencing according to the recommended parameters: AbSeq with 40,000 reads per cell, mRNA with 20,000 reads per cell, and sample tags with 600 reads per cell. The sequencing was performed on an Illumina NovaSeq using S1 and S2 100 cycle kits (Illumina, San Diego, CA, USA) with specific dimensions (67 × 8 × 50 bp). 

The resulting FASTA and FASTQ files were uploaded to the Seven Bridges Genomics pipeline (https://www.sevenbridges.com/apps-and-pipelines/, accessed on 9 November 2020), where data filtering was applied to generate matrices and CSV files. This analysis yielded draft transcriptomes and surface phenotypes of 213,515 cells involving 496 genes and 51 antibodies. After removing cell doublets based on sample tags and undetermined cells, 175,628 cells remained. Further doublets were eliminated using Doublet Finder (https://github.com/chris-mcginnis-ucsf/DoubletFinder, accessed on 7 December 2020), resulting in 162,454 remaining cells. Additionally, 291 NK cells were excluded as they appeared to be doublets with myeloid cells. NK cells were defined based on the presence of CD56+ and CD16−/+ and the absence of CD19-, CD3-, CD19-, CD4-, CD14-, and CD123- protein expression. 10494 NK cells (6.46% of total PBMCs) were successfully identified.

### 4.4. Thresholding and Clustering

Antibody thresholds were determined for each antibody by assessing its signal in negative cells or deconvoluting overlapping normal distributions of the known major cell types. All downstream analysis was performed in R version 4.3.2. The function normalmixEM from the mixtools R package was used to deconvolve the overlapping distributions. Ridgeline plots were used to set the best threshold for each antibody. Thresholding helps remove noise from non-specific antibody binding and enables characterizing cells with the right cell surface phenotype. Before clustering, the data were batch-corrected using the Harmony (v0.1.1) package. The dimensionality reduction of UMAP (Uniform Manifold Approximation and Projection) was used to project the cells onto a 2D space. The UMAP algorithm was applied to the first four principal components obtained from Harmony. The minimum distant parameter was set at 1. The Louvain clustering algorithm was used to cluster the cells based on their surface phenotypes. For NK cell clusters, NK cells were re-clustered based on the expressions of CD56, CD16, CD25, CD2, CD27, and HLA-DR. The resolution parameter was set to 0.08, and the random seed was set to 42 for the reproducibility of results. Five distinct populations were identified after clustering.

### 4.5. Single-Cell RNA-Seq Data Analysis

RNA and ADT quantifications for the identified NK cells were analyzed in R using Seurat (v4.3.0). Antibody data were CLR normalized and converted to the log_2_ scale, while transcripts were normalized based on total UMIs and converted to the log_2_ scale. Feature plots were generated using Seurat’s FeaturePlot function. Density plots were generated using the R package Nebulosa (v1.12.1). Differential expression or correlation analysis was performed on patients’ mean gene expression values to calculate the fold-change expression or correlation between the defined groups, *p*-values were calculated using patients’ mean gene expression values between the defined groups and tested by the Mann–Whitney test (two-tails or one-tail, as indicated in the figure legends), and plots were created using GraphPad 10. Heatmaps were generated using the heatmap (v1.0.12) R package and volcano plots using ggplot2 (v3.5.1). Gene expression data were scaled across all samples. Genes that were expressed in less than 40% of patients were removed to avoid misinterpretation of the results. Atherosclerotic plaque data were obtained from GSE23407 [31].

### 4.6. HCMV Serostatus ELISA

Anti-cytomegalovirus IgG1 serostatus was assessed using the human anti-cytomegalovirus IgG1 ELISA kit (CMV, Abcam, AB108724, Cambridge, UK). Patients’ serum samples were diluted at 1:10 and tested according to the company’s protocol.

### 4.7. Cytek or Flow-Cytometry Analysis

Frozen PBMC samples from the same 61 patients with or without CAD or diabetes were used to analyze NK cell subsets with a five-laser Cytek Aurora. A LIVE/DEAD™ Fixable Blue Dead Cell Stain Kit (Invitrogen, Waltham, MA, USA, cat. No: L34962) was used to exclude dead cells. AF647-conjugated anti-CD14 (BioLegend, San Diego, CA, USA, cat. No: 302046) and BV711-conjugated anti-CD1c (BioLegend cat. No: 331536) were used to exclude myeloid cells. BUV805-conjugated anti-CD3 (BioLegend cat. No: 612895) was used to exclude T cells, and PE/Fire 700-conjugated anti-CD19 (BioLegend cat. No: 302276) was used to exclude B cells. CD3-CD19- cells were gated using BV570-conjugatyed anti-CD56 (BioLegend cat. No: 362540) vs. BV785-conjugated anti-CD16 (BioLegend cat. No: 302046), and CD56+CD16-/+ lymphocytes were defined as NK cells as described in the figure legend. Percp-cy5.5-conjugated anti-NKG2A (BioLegend cat. No: 375126), Pacific-blue-conjugated anti-CD57 (BioLegend cat. No: 359608), PE-conjugated anti-NKG2C (BioLegend cat. No: 375004), FITC-conjugated anti-FcεR1γ (intracellular, Millipore Sigma, Burlington, MO, USA, cat. No: FCABS400F), BUV615- conjugated anti-NKG2D (BD™ Biosciences cat. No: 751232), PE-conjugated anti-human CD2 (BioLegend cat. No: 300207), and AF647-conjugated anti-human HLA-DR (BioLegend cat. No: 307621) were used for cell staining. Surface staining and intracellular staining were performed as previously described [22].

### 4.8. Primary NK Cell Culture and Spondin-2 Protein Expression

Human primary NK cells were isolated from Plateletpheresis leukoreduction filters (Vitalant, https://vitalant.org/Home.aspx) by using the negative selection “RosetteSep Human NK Cell Enrichment Cocktail” kit (#15065; STEMCELL Technologies, Vancouver, BC, Canada). Primary NK cells were cultured in CellGenix^®^ GMP stem-cell growth media (SCGM, 20802-0500, CellGenix, Freiburg, Germany) supplemented with 1% L-glutamine, 1% penicillin and streptomycin, 1% sodium pyruvate, 1% non-essential amino acids, 10 mM HEPES, and 10% human serum (heat-inactivated, sterile-filtered, male AB plasma; Sigma-Aldrich, St, Louis, MO, USA). Adaptive NK cell-positive donors were identified by detection of NKG2C (FAB138P or FAB138A antibodies; R&D Systems, Minneapolis, MN, USA) and FcεR1γ (FCABS400F antibody; Millipore-Sigma, Burlington, MA, USA) [22] protein expression on live (Zombie red, 77475; BioLegend), CD3-negative (300318 antibody; BioLegend), CD56^dim^ (318322 antibody; BioLegend), and CD16^+^ (302038 antibody; BioLegend) cells. NK cells were cultured for the indicated amount of time with or without human IL-2 (TECINTM; teceleukin; ROCHE, Basel, Switzerland), human IL-15 (247-IL/CF; R&D Systems), human TGFβ1 (580706; BioLegend), human IL-12 (219-IL; R&D Systems), D-(+)-Glucose (Millipore-Sigma, G7021), mTORC1 inhibitor (Calbiochem; rapamycin, 553210, IC50 = 0.1 µM), or FOXO1 inhibitor Calbiochem (AS1842856, 344355, IC50 = 33 nM). Surface or intracellular staining was performed as previously described [21]. Spondin-2 (Mindin Antibody (A-10) sc-166868 PE) and IFNγ (502516 antibody; BioLegend) were measured by intracellular cell staining. Primary NK cells anti-CD16 beads stimulation or cell proliferation detection using cell trance violet were performed as previously described [21]. Human TruStain FcX™ (Fc Receptor Blocking Solution, 422302; BioLegend) was used at 1:100 dilution for blocking nonspecific binding. Data acquisition was performed using an LSR-II flow cytometer, and analysis was performed by using FlowJo.v10 software.

### 4.9. Statistical Analysis

GraphPad Prism 10 or R statistical programming were used to calculate statistical differences using a Mann–Whitney test, one-tail, Pearson correlation, one-way ANOVA test, or chi-square test (one-tail) as described in the figure legends (* *p* ≤ 0.05; ** *p* < 0.01; *** *p* < 0.001). Data values represent patient mean gene expression. Unless otherwise indicated, all graphs show mean +/− population standard deviation (S.D.). Patient groups’ data points were integrated into one graph to allow better visualization of relative changes between HCMV^−^ and HCMV^+^ patients. A Mann–Whitney test comparing variables between patient groups was performed on HCMV^−^ patients (CAD/diabetes groups: number of tests = six) or HCMV^+^ patients (CAD/diabetes groups: number of tests = six) or between HCMV^−^ vs. HCMV^+^ patients within each defined patient group (CAD/diabetes groups: number of tests = four).

## Data Availability

All CAD and Atherosclerosis related data are available at GEO: GSE190570 and GSE23407.

## Supplementary Materials

The following supporting information can be downloaded at: https://www.mdpi.com/article/10.3390/ijms26031369/s1.
