# Supplementary material for: CITE-Seq Analysis Reveals a Differential Natural Killer Cell SPON2 Expression in Cardiovascular Disease Patients Impacted by Human-Cytomegalovirus Serostatus and Diabetes
Source: Int J Mol Sci. 2025 Feb 6;26(3):1369. doi: 10.3390/ijms26031369 (PMC11818894; doi:10.3390/ijms26031369)
Supplement: Supplementary file 1 [file ijms-26-01369-s001.zip › ijms-3410752-supplementary.pdf]

Supplementary Materials

Supplementary Table S1

	Diabetic$^-$	Diabetic$^+$	P value	P value (FDR)	CAD^{low}	CAD^{high}	P value	P value (FDR)
				CAD or diabetes patient groups				
Age	64.38 +/- 8.84	64.59 +/- 8.3	0.4326	0.515	63.72 +/- 8.12	65.15 +/- 8.97	0.2051	0.5905952
Sex (% Women)	20.58	37.04	0.1547	0.3223	24.14	31.25	0.5361	0.6092
Glucose (mg/dL)	97.73 +/- 13.93	140.70 +/- 42.89	<0.0001	0.00125	121.24 +/- 43.96	112.68 +/- 28.99	0.4929	0.5905952
A1c (%)	5.67 +/- 0.49	7.30 +/- 1.38	<0.0001	0.0013	6.58 +/- 1.52	6.23 +/- 0.96	0.405	0.5906
Gensini Score	2.18 +/- 2.57	2.46 +/- 2.19	0.3408	0.4484211	66.58 +/- 33.41	54.39 +/- 26.45	<0.0001	0.0025
CMV seropostivie	41.93548387	62.96296296	0.1099	0.2	44.82758621	62.5	0.1666	0.25
BMI	31.36 +6.43	33.48 + 6.33	0.1229	0.32225	34.18 + 6.53	30.58 + 5.92	0.0179	0.1991667
BP Systolic mmHG	136.52 +/- 14.68	142.07 +/- 24.16	0.2568	0.3666667	134.93 +/- 19.5	142.65 +/- 19.05	0.0418	0.26125
BP Diastolic mmHG	79.14 +/- 14.02	74.74 +/- 11.43	0.1532	0.3222917	78.10 +/- 12.33	76.37 +/- 13.75	0.2841	0.5905952
Creatinine (mg/dL)	0.90 +/- 0.18	0.72 +/- 0.23	0.0024	0.02	0.80 +/- 0.24	0.84 +/- 0.2	0.4178	0.5905952
hsCRP (mg/L)	3.07 +/- 3.53	8.31 +/- 27.88	0.2386	0.3666667	3.30 +/- 3.64	7.28 +/- 25.74	0.4871	0.5905952
Total Cholesterol (mg/dL)	149.76 +/- 40.34	143.03 +/- 35.75	0.2569	0.3666667	141.34 +/- 26.54	151.71 +/- 46.25	0.3218	0.5905952
Triglycerides (mg/dL)	102.88 +/- 47.49	153.07 +/- 85.74	0.0124	0.06041667	136.96 +/- 75.06	114.34 +/- 66.59	0.0945	0.39375
HDL Cholesterol (mg/dL)	43.08 +/- 11.62	40.14 +/- 14.49	0.0782	0.2792857	39.65 +/- 10.85	43.71 +/-14.49	0.1778	0.5905952
LDL Cholesterol (mg/dL)	89.58 +/- 33.1	77.55 +/- 30.25	0.1289	0.32225	78.89 +/- 23.08	89.12 +/- 38.36	0.3015	0.5905952
Smoking current (% positive)	8.82	7.41	0.8413	0.8763542	6.90	9.38	0.7245	0.7875
Smoking former (% positive)	41.18	55.56	0.264	0.3666667	55.17	40.63	0.2559	0.5905952
Statin (% positive)	70.59	96.30	0.0095	0.059375	82.76	81.25	0.8784	0.8784
HTN- Diuretics (% positive)	26.47	22.22	0.7019	0.7629348	13.79	34.38	0.0623	0.3115
HTN- Beta Blockers (% positive)	47.06	66.67	0.1943	0.3666667	62.07	50.00	0.4408	0.5905952
HTN- Calcium Channel Blockers (% positive)	20.59	22.22	1	1	13.79	28.13	0.2192	0.5905952
HTN- ACE (% positive)	26.47	48.15	0.1091	0.32225	37.93	34.38	0.7957	0.8288542
HTN- Angiotensin II Receptors (% positive)	5.88	14.81	0.4011	0.501375	13.79	6.25	0.4107	0.5905952
NSAIDS (% positive)	79.41	88.89	0.4892	0.5559091	79.31	87.50	0.4961	0.5905952
Patinets #	34	27			29	32		

Supplementary Table S2

	0-6	31-48	49-69.6	77-150	P value	P value (FDR)
			Stenosis severity score (patient groups)			
Age	63.72+/-8.2	67.84+/-8.6	60.45+/-9.9	67.25+/-6.7	0.1467	0.4500
Sex (% Women)	24.14	69.23	9.09	0.00	0.001	0.012
Glucose (mg/dL)	121.24+/-44.7	111.84+/-25.4	109.18+/-28	118.87+/-39.25	0.782	0.8531
A1c (%)	6.58+/-1.55	6.43+/-0.9	6.04+/-0.8	6.17+/-1.3	0.65	0.7657
Gensini Score	67	37.03846154	55.90909091	107.9375	<0.0001	0.0024
CMV seropostivie	44.83	61.54	72.73	50.00	0.41	0.66
BMI	34.18965517	29.16923077	32.17272727	30.70875	0.1144	0.4500
BP Systolic mmHG	134.93+/-19.8	147.30+/-25.1	140+/-11.4	138.75+/-17.9	0.32	0.62
BP Diastolic mmHG	78.10+/-12.5	76+/-12.1	77.72+/-14.4	75.12+/-17.4	0.9	0.9000
Creatinine (mg/dL)	0.80+/-0.25	0.75+/-0.15	0.91+/-0.19	0.87+/-0.25	0.21	0.5600
hsCRP (mg/L)*	3.30+/-3.7	3.07+/-2.33	2.48+/-2.26	2.34+/-3.2	0.82	0.8557
Total Cholesterol (mg/dL)	141.34+/-27.01	162.30+/-48.05	156.63+/-38.84	127.75+/-52.63	0.15	0.4500
Triglycerides (mg/dL)	136.96+/-76.39	103.07+/-54.3	150.45+/-81.57	83+/-48.02	0.1	0.4500
HDL Cholesterol (mg/dL)	39.65+/-11.04	49.92+/-19	38.72+/-9.1	40.5+/-10	0.09	0.4500
LDL Cholesterol (mg/dL)	78.89+/-23.4	95.38+/-39	93+/-36.3	73.62+/-43	0.27	0.6199
Smoking current (% positive)	6.90	15.38	9.09	0.00	0.64	0.77
Smoking former (% positive)	55.17	30.77	36.36	62.50	0.3323	0.6199
Statin (% positive)	82.76	84.62	90.91	62.50	0.44	0.66
HTN- Diuretics (% positive)	13.79	23.08	36.36	50.00	0.1418	0.4500
HTN- Beta Blockers (% positive)	62.07	53.85	54.55	37.50	0.67	0.77
HTN- Calcium Channel Blockers (% positive)	13.79	38.46	18.18	25.00	0.3358	0.6199
HTN- ACE (% positive)	37.93	38.46	18.18	50.00	0.52	0.73
HTN- Angiotensin II Receptors (% positive)	13.79	7.69	0.00	12.50	0.6055	0.7657
NSAIDS (% positive)	79.31	84.62	100.00	75.00	0.39	0.66
Patinets #	29	13	11	8		

Supplementary Table S3

	Diabetic$^-$ CAD^{low}	Diabetic$^+$ CAD^{low}	Diabetic$^-$ CAD^{high}	Diabetic$^+$ CAD^{high}	P value	P value (FDR)
			CAD and diabetes patient groups			
Age	64.06 +/- 8.4	63.30 +/- 7.74	64.66 +/- 9.21	65.78 +/- 8.62	0.9018	0.9018
Sex (% Women)	18.75	30.76	22.22	42.85	0.1248	0.2836
Glucose (mg/dL)	99.5 +/- 16.27	148 +/- 51.84	96.16 +/- 11.23	133.92 +/- 30.94	<0.0001	0.0008
A1c (%)	5.71 +/- 0.6	7.65 +/- 1.63	5.65 +/- 0.36	6.98 +/- 0.98	<0.0001	0.0008
Gensini Score	2.18 +/- 2.57	2.46 +/- 2.19	66.58 +/- 33.41	54.39 +/- 26.45	<0.0001	0.0008
CMV seropostivie	50.00	38.46	44.44	85.71	0.05	0.25
BMI	33.19 +/- 6.86	35.41 +/- 5.88	29.73 +/- 5.53	31.68 +/- 6.21	0.101	0.2836364
BP Systolic mmHG	132.06 +/- 17.66	138.46 +/- 21.02	140.5 +/- 9.82	145.42 +/- 26.30	0.32	0.49
BP Diastolic mmHG	78.68 +/- 12.85	77.38 +/- 11.62	79.55 +/- 14.96	72.28 +/- 10.69	0.4484	0.58275
Creatinine (mg/dL)	0.90 +/- 0.15	0.68 +/- 0.28	0.9 +/- 0.2	0.76 +/- 0.17	0.02	0.10
hsCRP (mg/L)	3.78 +/- 4.26	2.72 +/-2.56	2.439444444	13.51071429	0.3494	0.4943056
Total Cholesterol (mg/dL)	142.87 +/- 26.32	139.46 +/- 26.68	155.88 +/- 48.76	146.35 +/- 42.19	0.67	0.72
Triglycerides (mg/dL)	113.37 +/- 47.78	166 +/- 90.73	93.55 +/-45.24	141.07 +/- 78.96	0.0291	0.1455
HDL Cholesterol (mg/dL)	40.87 +/- 11.90	38.15 +/- 9.18	45.05 +/- 11	42 + 17.87	0.55	0.65
LDL Cholesterol (mg/dL)	83.06 +/-21.38	73.76 +/- 24.04	95.38 +/- 39.90	81.07 +/- 34.67	0.3163	0.4943056
Smoking current (% positive)	0.00	7.69	11.11	7.14	0.61	0.70
Smoking former (% positive)	50.00	61.54	33.33	50.00	0.4662	0.58275
Statin (% positive)	38.46	0.00	27.78	7.14	0.07	0.25
HTN- Diuretics (% positive)	12.50	15.38	38.89	28.57	0.1228	0.2836364
HTN- Beta Blockers (% positive)	50.00	76.92	44.44	57.14	0.25	0.48
HTN- Calcium Channel Blockers (% positive)	18.75	7.69	22.22	35.71	0.3559	0.4943056
HTN- ACE (% positive)	25.00	46.15	22.22	50.00	0.25	0.48
HTN- Angiotensin II Receptors (% positive)	12.50	15.38	0.00	14.29	0.3518	0.4943056
NSAIDS (% positive)	68.75	92.31	88.89	85.71	0.12	0.28
Patinets #	16	13	18	14		

Supplementary Table S1: Clinical data of patients grouped by CAD or diabetes status. Data are presented as mean +/- S.D or percentage of the population. Statistical analysis was done using the chi-square or Mann-Whitney test, one-tail. p >0.05 was considered significant.

Supplementary Table S2: Clinical data of patients grouped by stenosis severity. Data are presented as mean +/- S.D or percentage of the population. Statistical analysis was done using the chi-square or one-way ANOVA tests. p >0.05 was considered significant.

Supplementary Table S3: Clinical data of patients grouped by CAD and diabetes status. Data are presented as mean +/- S.D or percentage of the population. Statistical analysis was done using the chi-square or one-way ANOVA tests. p >0.05 was considered significant.

Figure S1

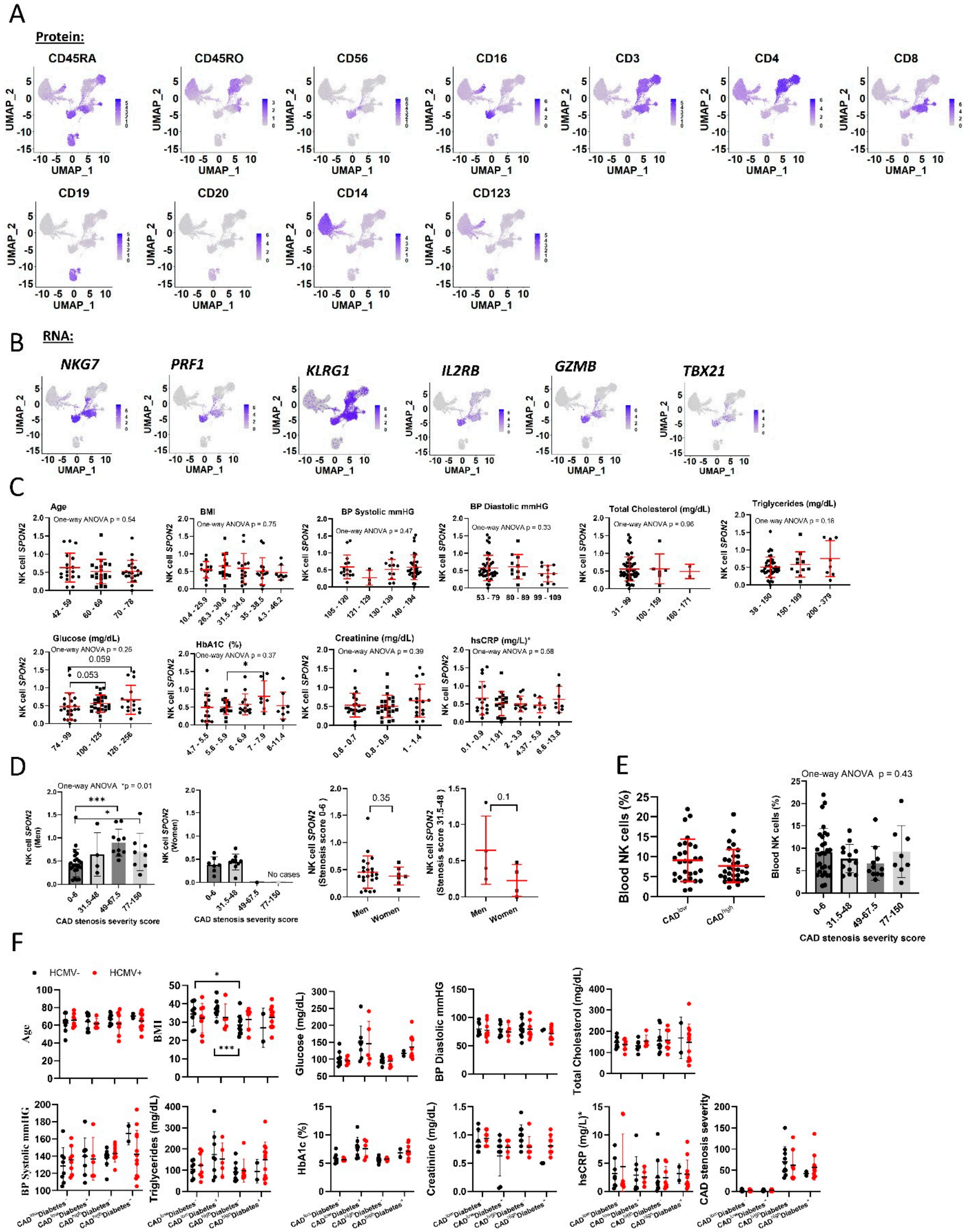
A
Protein:
CD45RA
CD45RO
CD56
CD16
CD3
CD4
CD8
CD19
CD20
CD14
CD123
UMAP_1
UMAP_2
B
RNA:
NKG7
PRF1
KLRG1
IL2RB
GZMB
TBX21
C
Age
One-way ANOVA p = 0.54
BMI
One-way ANOVA p = 0.75
BP Systolic mmHG
One-way ANOVA p = 0.47
BP Diastolic mmHG
One-way ANOVA p = 0.33
Total Cholesterol (mg/dL)
One-way ANOVA p = 0.96
Triglycerides (mg/dL)
One-way ANOVA p = 0.16
Glucose (mg/dL)
One-way ANOVA p = 0.25
0.059
0.053
HbA1C (%)
One-way ANOVA p = 0.37
Creatinine (mg/dL)
One-way ANOVA p = 0.39
hsCRP (mg/L)*
One-way ANOVA p = 0.58
NK cell SPON2
D
One-way ANOVA *p = 0.01
NK cell SPON2 (Men)
NK cell SPON2 (Women)
No cases
CAD stenosis severity score
0.35
NK cell SPON2 (Stenosis score 0-6)
0.1
NK cell SPON2 (Stenosis score 31.5-48)
Men
Women
E
Blood NK cells (%)
One-way ANOVA p = 0.43
CAD stenosis severity score
F
HCMV-
HCMV+
Age
BMI
Glucose (mg/dL)
BP Diastolic mmHG
Total Cholesterol (mg/dL)
BP Systolic mmHG
Triglycerides (mg/dL)
HbA1c (%)
Creatinine (mg/dL)
hsCRP (mg/L)*
CAD stenosis severity

Supplementary Figure S1: PBMC clusters classification and patient groups variation-associated clinical parameters. A) UMAP of PBMC clusters for the specific CITE-seq protein markers CD45RA, CD45RO, CD56, CD16, CD3, CD4, CD8, CD19, CD20, CD14, or CD123. **B)** Single-cell RNA-seq analysis of the relevant NK cell-associated gene markers, *NKG7, PRF1, GZMB, KLRG1, IL12RB,* and *TBX21.* **C**) NK cell *SPON2* expression relative to the indicated clinical parameters. **D**) NK cell *SPON2* expression in men or women. **E)** Blood NK cell frequencies between CAD^{low} vs. CAD^{high} patients (left) or relative to stenosis severity (right). **F)** Variation of clinical parameters between patients grouped by CAD, diabetes, and HCMV status. Mean+/- S.D., Mann-Whitney test, one-tail, *p < 0.05, ** p < 0.01, *** p < 0.001. dot = patient. *To avoid misinterpretation of the data, one patient outlier (hs-CRP (mg/L) = 150) was removed from the analysis

Figure S2

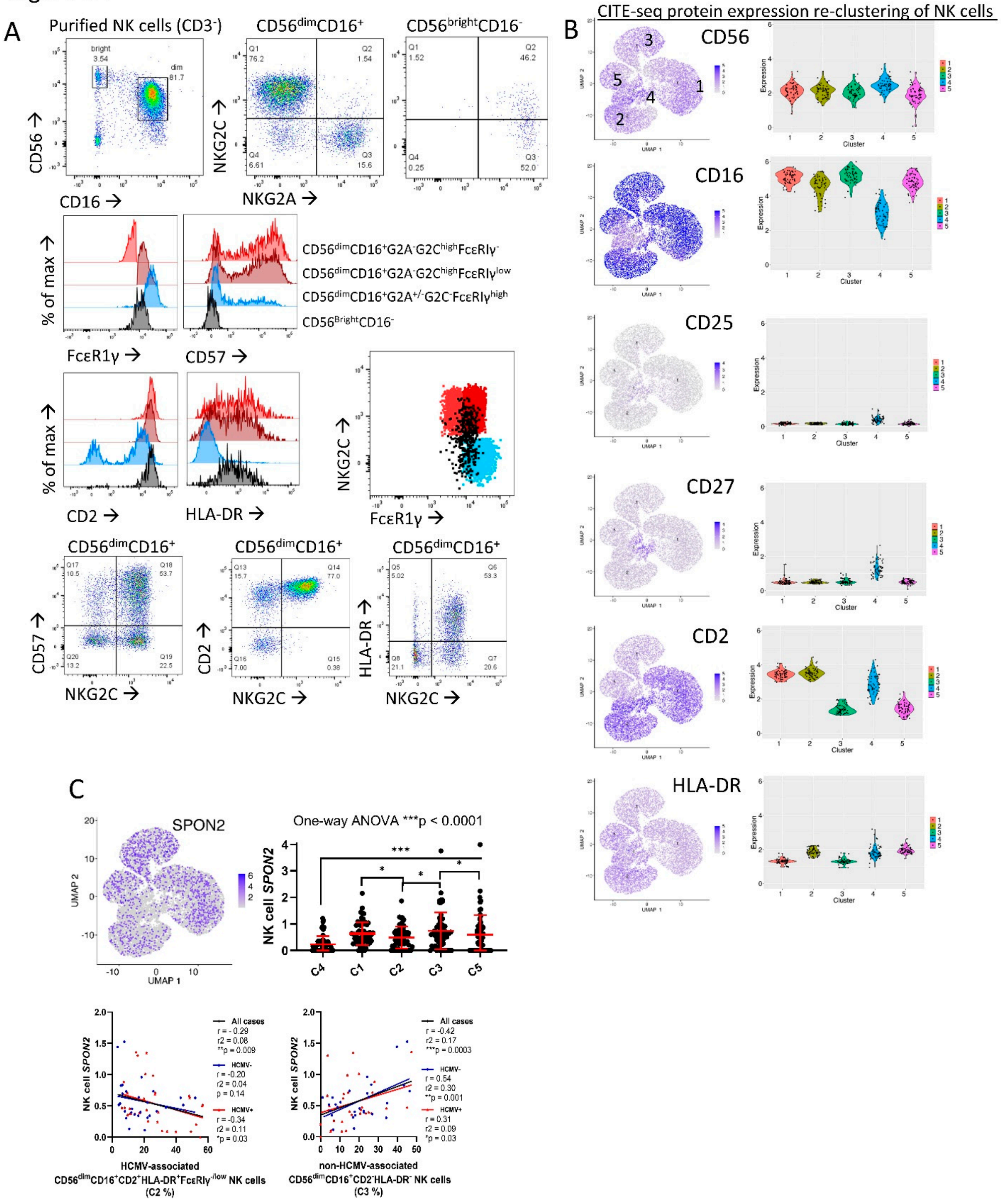

A
Purified NK cells (CD3-)
CD56dimCD16+
CD56brightCD16-
bright 3.54
dim 81.7
CD56 →
CD16 →
NKG2C →
NKG2A →
Q1 76.2
Q2 1.54
Q4 6.61
Q3 15.6
Q1 1.52
Q2 46.2
Q4 0.25
Q3 52.0
% of max →
FcεR1γ →
CD57 →
CD56dimCD16+G2A-G2ChighFcεRIγ-
CD56dimCD16+G2A-G2ChighFcεRIγlow
CD56dimCD16+G2A+/-G2C-FcεRIγhigh
CD56BrightCD16-
CD2 →
HLA-DR →
NKG2C →
FcεR1γ →
CD56dimCD16+
CD57 →
NKG2C →
CD2 →
HLA-DR →
Q17 10.5
Q18 53.7
Q20 13.2
Q19 22.5
Q13 15.7
Q14 77.0
Q16 7.00
Q15 0.38
Q5 5.02
Q6 53.3
Q8 21.1
Q7 20.6
B
CITE-seq protein expression re-clustering of NK cells
CD56
CD16
CD25
CD27
CD2
HLA-DR
UMAP 1
UMAP 2
Expression
Cluster
C
SPON2
One-way ANOVA ***p < 0.0001
NK cell SPON2
C4
C1
C2
C3
C5
All cases
r = -0.29
r2 = 0.08
**p = 0.009
HCMV-
r = -0.20
r2 = 0.04
p = 0.14
HCMV+
r = -0.34
r2 = 0.11
*p = 0.03
HCMV-associated
CD56dimCD16+CD2+HLA-DR+FcεRIγ-/low NK cells
(C2 %)
All cases
r = -0.42
r2 = 0.17
***p = 0.0003
HCMV-
r = 0.54
r2 = 0.30
**p = 0.001
HCMV+
r = 0.31
r2 = 0.09
*p = 0.03
non-HCMV-associated
CD56dimCD16+CD2-HLA-DR- NK cells
(C3 %)

Supplementary Figure S2: NK cell clusters classification. A) Representative flow cytometry staining of human NK cells from adaptive NK cell positive donor for FcεR1γ relative to CD2, HLA-DR, or CD57 expression between immature $CD56^{bright}CD16^{-}$ (black), mature $CD56^{dim}CD16^{+}$ - $NKG2A^{+}NKG2C^{-}$ (blue), $NKG2A^{-/+}NKG2C^{-}$ (blue), and adaptive $NKG2A^{-}NKG2C^{high}Fc\varepsilon R1\gamma^{low}$ (dark red) or $Fc\varepsilon R1\gamma^{-}$ (light red). **B)** CITE-seq protein expression-based re-clustering. UMAP of NK cell clusters for the expression of CITE-seq protein markers: CD56, CD16, CD25, CD27, CD2, and HLA-DR. right: Violin plots of relative expression between the NK cell clusters. **C)** UMAP of NK cell clusters for *SPON2* expression (upper left), NK cell *SPON2* expression relative to each NK cluster, dot = patient (upper right), NK cell *SPON2* expression correlation to C2 (lower left) or C3 (lower right) frequencies (black: all patients, blue: $HCMV^{-}$ patients, or red: $HCMV^{+}$ patients. Mean+/- S.D., Mann-Whitney test, one-tail, *p < 0.05, ** p < 0.01, *** p < 0.001. or Pearson correlation (one-tail).

Figure S3

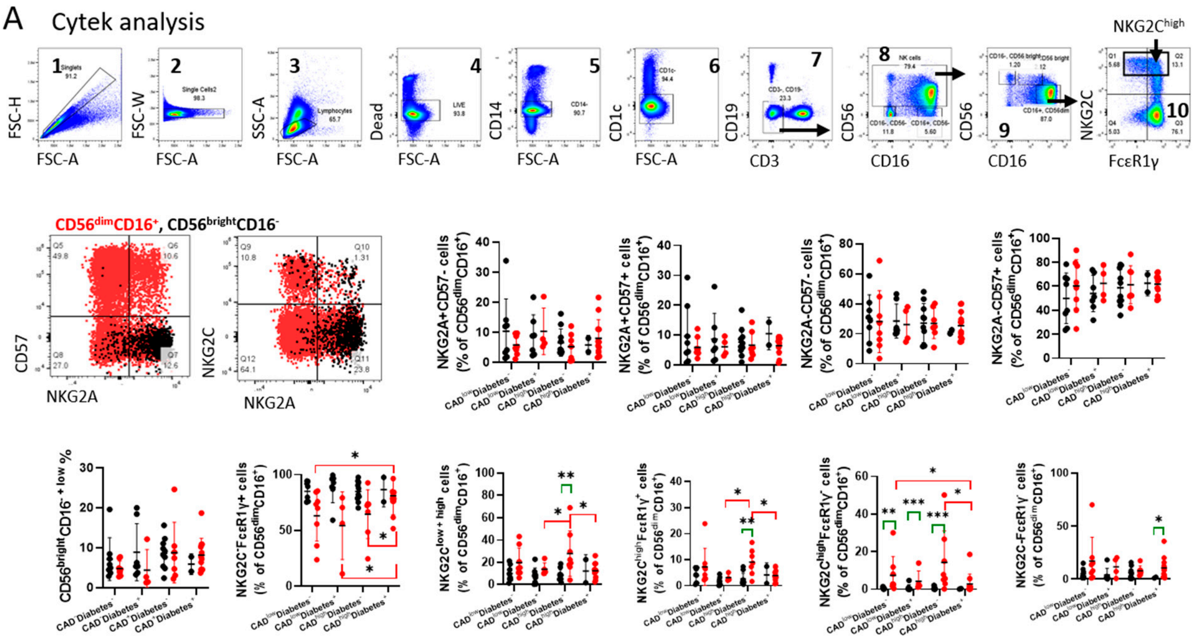

Supplementary Figure S3: Cytek analysis of CAD PBMC samples. A) Upper panels: Cytek analysis of CAD cohort patients' PBMC (n = 61), relative to CAD, diabetes, and HCMV status. Gating strategy of PBMC: PBMC were gated to exclude doublets by FSC-A vs. FSC-H [1] and FSC-A vs. FSC-W [2], followed by generating a lymphocyte gate [3] and removing of dead cells [4], $CD14^{+}$ cells [5], and $CD1c^{+}$ cells [6]. The remaining cells were then plotted by CD3 (T cells) vs. CD19 (B cells) [7], and $CD3^{-}CD19^{-}$ cells were plotted by CD56 vs. CD16 to identify NK cells [8]. NK cells were then gated as immature $CD56^{bright}CD16^{-}$, immature $CD56^{bright}$ $CD16^{low}$, and mature $CD56^{dim}CD16^{+}$ [9]. Mature NK cells were plotted by FcεR1γ vs. NKG2C to identify adaptive NK cell subsets [10]. **Middle panels:** left to right: dot plots of NKG2A vs. CD57 or NKG2A vs. NKG2C in mature $CD56^{dim}CD16^{+}$ (red) vs. immature $CD56^{bright}CD16^{-}$ (black), percentages of NKG2A vs. CD57 mature NK cell subsets between patients grouped by CAD, diabetes, or HCMV status. **Lower panels:** left to right: immature ($CD56^{bright}CD16^{-/low}$), non-adaptive mature ($NKG2C^{-}FcεR1γ^{+}$ or $NKG2C^{-}FcεR1γ^{-}$), or adaptive mature $NKG2C^{low+high}$, $NKG2C^{high}FcεR1γ^{+}$, or $NKG2C^{high}FcεR1γ^{-}$ NK cell subset percentages between patients grouped by CAD, diabetes, or HCMV status. Statistical analysis of panels C, D, and F, Mean+/- S.D, Mann, Whitney test, one-tail (* p <0.05, **

p <0.01, ***p <0.001, dot = patient, green bars: $HCMV^-$ vs. $HCMV^+$, Black bars: between $HCMV^-$ patients' groups, red bars: between $HCMV^+$ patients' groups). Note that $CD56^{dim}CD16^{low/-}$ cells were not included in the analysis as reduced CD16 expression is associated with NK cell activation (caused by ADAM17) which can lead to lower protein expression of the adaptor protein FcεR1γ[22].

Figure S4

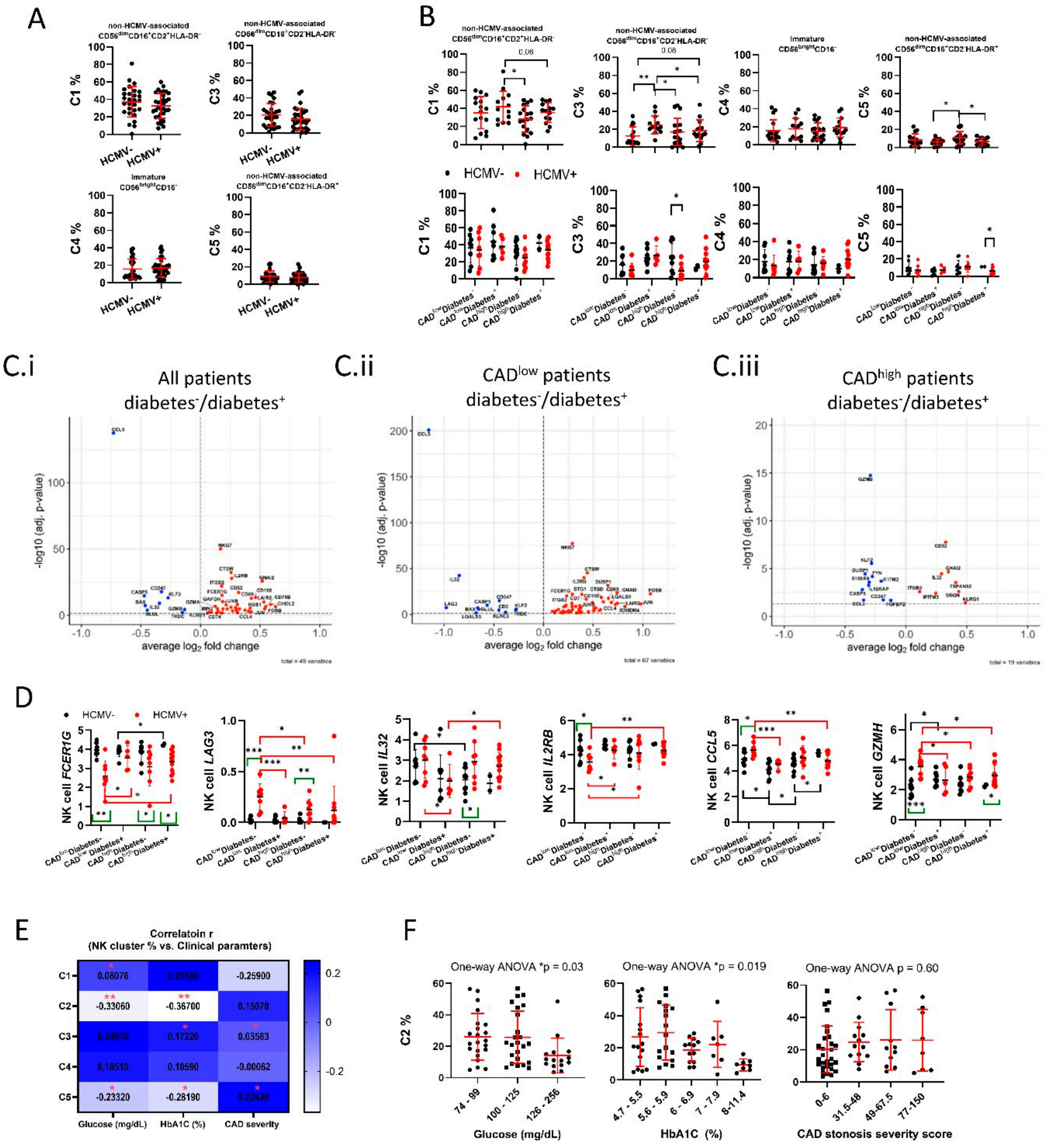
A
B
C.i
All patients diabetes-/diabetes+
C.ii
CADlow patients diabetes-/diabetes+
C.iii
CADhigh patients diabetes-/diabetes+
D
E
Correlatoin r (NK cluster % vs. Clinical paramters)
F
One-way ANOVA *p = 0.03
One-way ANOVA *p = 0.019
One-way ANOVA p = 0.60
Glucose (mg/dL)
HbA1C (%)
CAD stonosis severity score

Supplementary Figure S4: impact of diabetes on CITE-seq NK cell clusters. C1, C3, C4, or C5 frequencies between A**)** HCMV^{-} vs. HCMV^{+} patients. **B)** Patients grouped by CAD, diabetes, and HCMV status. **C)** DGE analysis between patients, group by diabetes status, C.i) all patients, C.ii) CAD^{low} patients, and C.iii) CAD^{high} patients. Increase expression in diabetes^{+} patients (red), or diabetes^{-} patients (blue). **D)** Mean expression of *FCER1G*, *LAG3*, *IL32*, *IL2RB*, *CCL5*, or *GZMH* in patients, grouped by CAD, diabetes, and HCMV status. **E)** Heatmap showing person correlation r values between NK cell clusters (C1- C5) frequencies and glucose (mg/mL), HbA1c %, or stenosis severity (one-tails, *p<0.05, **p<0.01). **F)** Comparison of C2 cluster frequencies between patients grouped by (left to right): glucose (mg/mL), HbA1c %, or stenosis severity (one-way ANOVA). Statistical analysis: person correlation (one-tail) or mean+/- S.D, Mann-Whitney test, one-tail (* p <0.05, ** p <0.01, ***p <0.001, dot = patient.

Figure S5

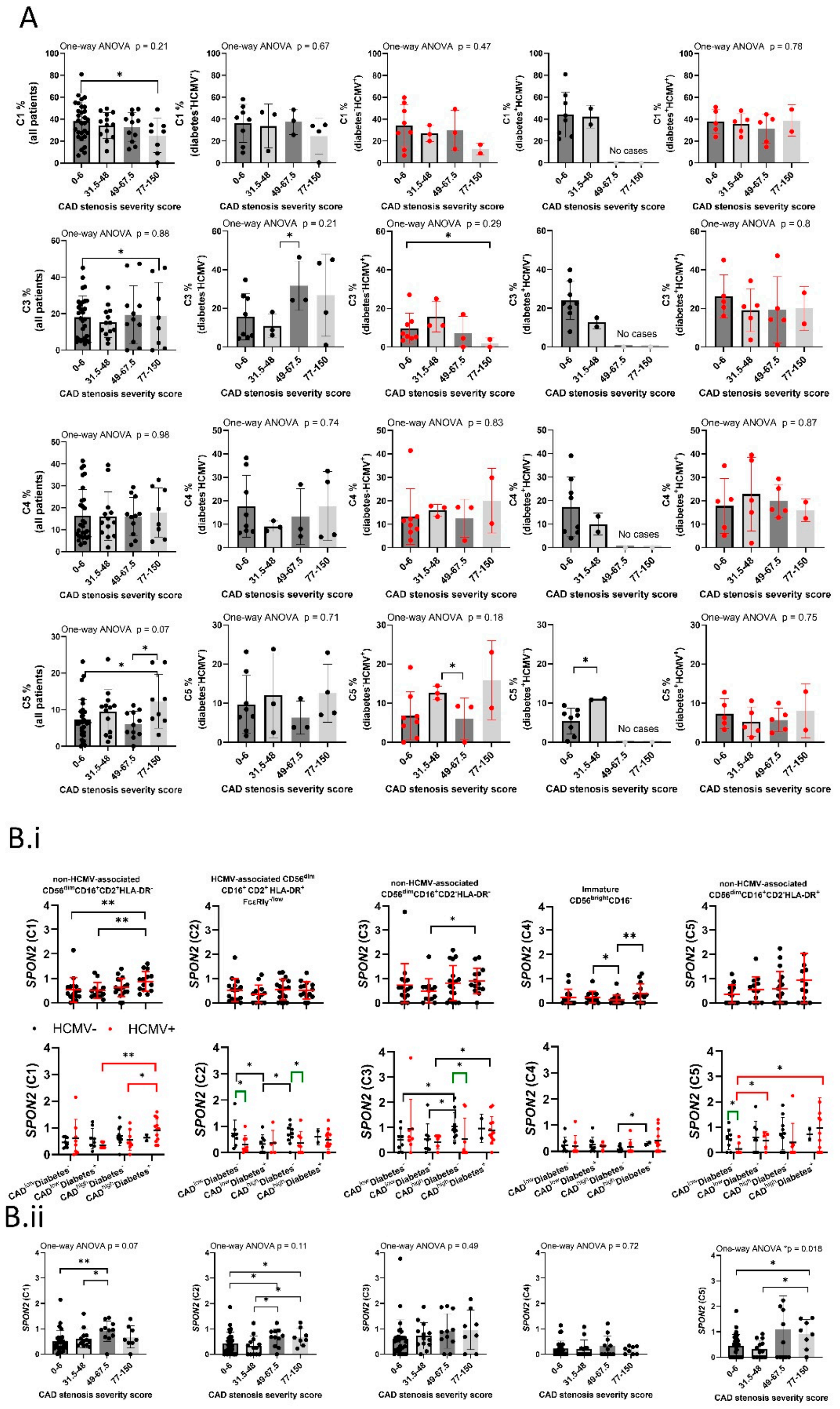

Supplementary Figure S5: Frequencies of NK cell clusters relative to stenosis severity. A) Frequencies of NK cell clusters relative to stenosis severity in patients, grouped by diabetes and HCMV status. **B)** *SPON2* expression in C1, C2, C3, C4, or C5, in .i) patients grouped by CAD, diabetes, and HCMV status, or .ii) relative to stenosis severity. Mean+/- S.D, Mann-Whitney test, one-tail, * p <0.05, *p<0.01, dot = patient.

Figure S6

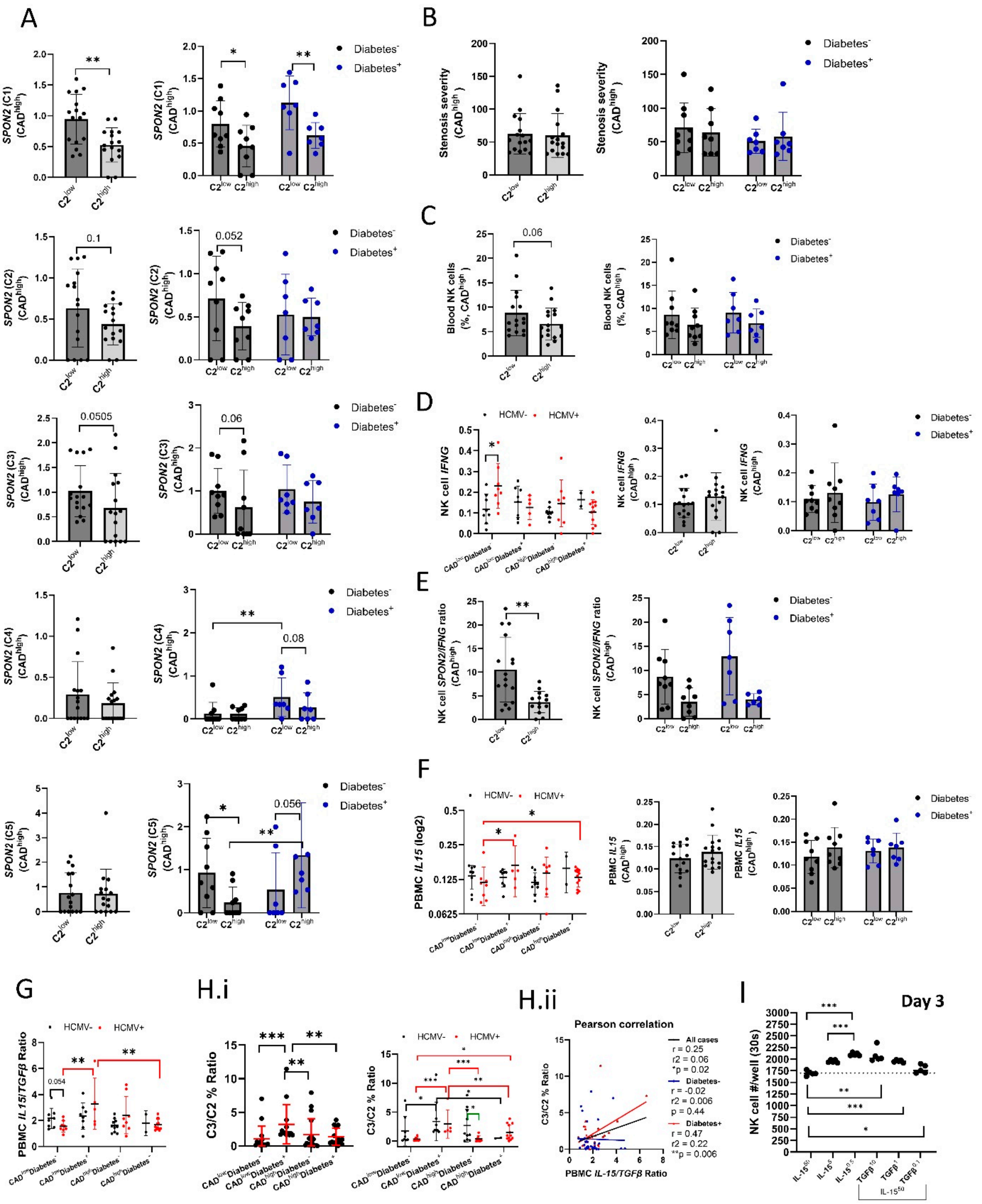
A
B
C
D
E
F
G
H.i
H.ii
I
Diabetes-
Diabetes+
HCMV-
HCMV+
SPON2 (C1) (CAD^high)
SPON2 (C2) (CAD^high)
SPON2 (C3) (CAD^high)
SPON2 (C4) (CAD^high)
SPON2 (C5) (CAD^high)
Stenosis severity (CAD^high)
Blood NK cells (%, CAD^high)
NK cell IFNG
NK cell IFNG (CAD^high)
NK cell SPON2/IFNG ratio (CAD^high)
PBMC IL15 (log2)
PBMC IL15 (CAD^high)
PBMC IL15/TGFβ Ratio
C3/C2 % Ratio
Pearson correlation
All cases
r = 0.25
r2 = 0.06
*p = 0.02
Diabetes-
r = -0.02
r2 = 0.006
p = 0.44
Diabetes+
r = 0.47
r2 = 0.22
**p = 0.006
PBMC IL-15/TGFβ Ratio
Day 3
NK cell #/well (30s)
IL-15^50
C2^low
C2^high
0.1
0.052
0.0505
0.06
0.08
0.056
0.054

Supplementary Figure S6: *SPON2* expression increase in all NK cell clusters relative to stenosis severity. A) Mean *SPON2* expression in C1, C2, C3, C4, or C5 NK cells in CAD^{high} patients grouped by $C2^{low}$ vs. $C2^{high}$ (left), and relative to diabetes status (right). **B)** CAD stenosis, or **C)** blood NK cell frequencies in CAD^{high} $C2^{low}$ or $C2^{high}$ patients (left), and relative to diabetes status (right, black: $diabetes^{-}$, blue: $diabetes^{+}$). **D)** Mean NK cell *IFNG* expression in (left) patients grouped by CAD, diabetes, and HCMV status, or (right) in CAD^{high} patients grouped by $C2^{low}$ vs. $C2^{high}$ (left), and relative to diabetes status (right). **E)** NK cell *SPON2*/*IFNG* ratio in CAD^{high} $C2^{low}$ or $C2^{high}$ patients (left), and relative to diabetes status (right, black: $diabetes^{-}$, blue: $diabetes^{+}$). **F)** Mean PBMC *IL15* expression in left; patients grouped by CAD, diabetes, and HCMV status, or right: in CAD^{high} patients grouped by $C2^{low}$ vs. $C2^{high}$ (left), and relative to diabetes status (right). **G)** Mean PBMC *IL15* expression in patients grouped by CAD, diabetes, and HCMV status. **H)** C3/C2 frequencies ratio in patients grouped by CAD, diabetes, and HCMV status (H.i), and Person correlation (one-tail) between PBMC *IL15*/*TGFβ* ratio and C3/C2 C3/C2 frequencies ratio, in all (black), $diabetes^{-}$ (blue, or $diabetes^{+}$ (red) patients (H.ii). **I)** Numbers of NK cells per well following 3 days of stimulation with the indicated IL-15 concentration with or without TGFβ. Mean+/- S.D, Mann Whitney test, one-tail (* p <0.05, *p < 0.01, *p < 0.001), dot = patient or donor.
